# Gold(I)-catalyzed hydroarylation reaction of aryl (3-iodoprop-2-yn-1-yl) ethers: synthesis of 3-iodo-2*H*-chromene derivatives

**DOI:** 10.3762/bjoc.9.249

**Published:** 2013-10-16

**Authors:** Pablo Morán-Poladura, Eduardo Rubio, José M González

**Affiliations:** 1 Departamento de Química Orgánica e Inorgánica and Instituto Universitario de Química Organometálica “Enrique Moles”, Universidad de Oviedo, C/Julián Clavería 8, Oviedo, 33006, Spain

**Keywords:** alkyne, chromene, gold, gold catalysis, hydroarylation, iodine

## Abstract

An efficient entry to the preparation of elusive 4-unsubstituted-3-iodo-2*H*-chromenes has been accomplished as result of a catalytic cyclization. Thus, upon exposition of [(3-iodoprop-2-yn-1-yl)oxy]arenes to IPrAuNTf_2_ (3 mol %), in 1,4-dioxane at 100 °C, the desired heterocyclic motif is readily assembled. This process nicely tolerates a variety of functional groups and, interestingly, it is compatible with the presence of strong electron-withdrawing groups attached to the arene. The overall transformation can be termed as a new example of a migratory cycloisomerization and, formally, it involves well-blended 1,2-iodine shift and hydroarylation steps.

## Introduction

The structure of 2*H*-chromene embodies a relevant heterocyclic motif, which is present in naturally occurring compounds [1–6] and encodes interesting properties that renders it attractive for functional applications. Thus, for instance, this molecular frame has been associated with photochromic crystals [7], photochromic organogelators [8], selective cyclooxygenase-2 inhibitors [9–10], antifungal [11] and antitrypanocidal activity [12], transforming growth factor-β receptors [13] and with potential novel termiticides [14], among other remarkable applications. On this basis, new approaches to access this relevant heterocyclic scaffold are the subject of ongoing synthetic interest [15–22].

**Scheme 1:**
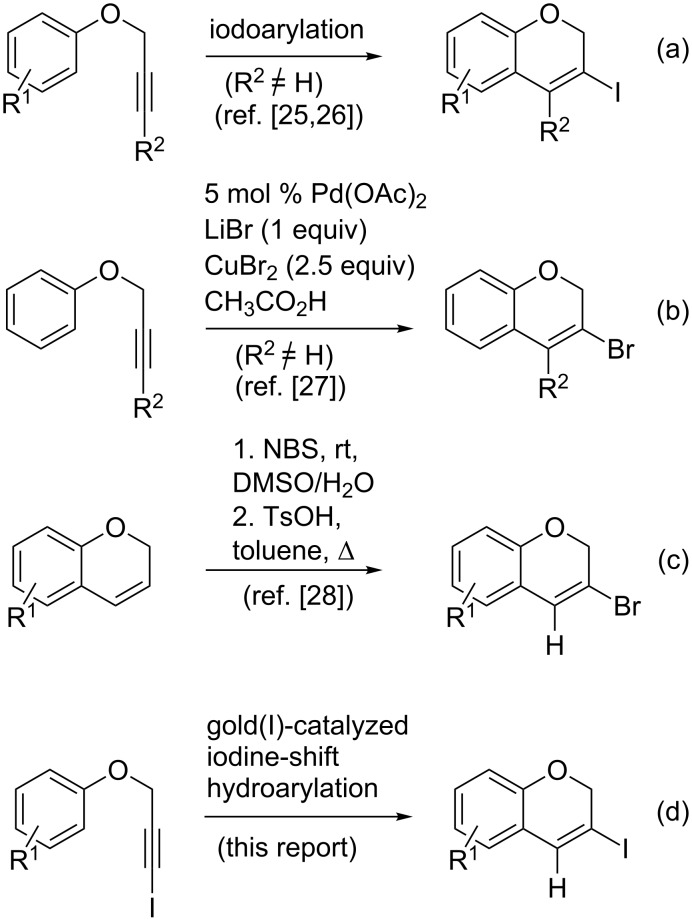
Synthesis of 3-halo-2*H*-chromenes.

In connection with synthetic efforts searching for new Hsp90 inhibitors [23], the metal-catalyzed coupling reaction of nitrogen-containing nucleophiles with 3-halo-substituted chromenes has been recognized as a convenient synthetic tool, which provides smooth access to potentially useful candidates [24]. The required halogen containing building-blocks can be prepared from aryl propargyl ethers following either metal-free iodinating [25–26] or a palladium-catalyzed brominating [27] approach that yield the required halogenated regioisomer at the time of assembling the target heterocyclic skeleton. These strategies are quite general to give 3-halo-4-substituted-2*H*-chromenes (see Scheme 1, entries a and b, respectively). However, they fail to produce simple 3-halo-4-unsubstituted derivatives. This synthetic context suggests a timely opportunity for devising new protocols to access the latter class of 3-halo-2*H*-chromene scaffolds from readily available precursors. Although a stepwise selective modification of the preassembled heterocycle has been recently developed (Scheme 1 entry c) [28], a de novo elaboration of 3-halo-2*H*-chromenes giving straight access to the desired regioisomer is yet to be accomplished. A desirable approach would also consider the generation of an increase in the molecular diversity, offering a suitable strategy intended for library discovery.

It is well known that metal-catalyzed hydroarylation is a powerful reaction to prepare benzofused heterocyclic compounds [29–31]. Pt(IV) [32], Pt(II) and Au(I) [33] complexes were early recognized as suitable catalysts to convert aryl propargyl ethers into chromenes. Nowadays, alternative gold(I)-based catalysts have been successfully exploited to further prepare substituted 2*H*-chromenes [34–38], as well as a wide variety of relevant heterocyclic compounds [39–40].

On the other hand, migratory cycloisomerization are important processes in contemporary catalysis [41]. In this context, our group is interested in C–H functionalization reactions of arenes involving propargylic derivatives [42]. Furthermore, the influence of different gold(I) catalysts over the outcome of the cyclization of *N*-(3-iodoprop-2-ynyl)-*N*-tosylanilines has been noticed [43]. For the latter reaction, conditions to modulate the relative amount of each of the possible regioisomeric cyclization products formed, with or without iodine shift, were outlined. Tuning the ligand at the gold atom [44] was used to accomplish a reaction manifold earlier recognized in the synthesis of regioisomeric halogenated phenanthrenes, but they are using two different metals [45]. Catalytic cycloisomerization reactions of heteroatom-substituted alkynes that take place without heteroatom migration are known [46–48].

On this ground, we were curious about the attractive possibility of combining known reaction profiles in an attempt to execute an efficient entry into the elusive 4-unsubstituted-3-iodo-2*H*-chromene derivatives. We hypothesize that this specific heterocyclic motif can be conveniently prepared from cyclization of aryl (3-iodoprop-2-yn-1-yl) ethers relying on the power of gold(I) catalysis, as depicted in Scheme 1 entry d. Herein, we report a new strategy to carry out this transformation.

## Results and Discussion

In a previous work on gold-catalyzed cyclization reactions of *N*-(3-iodoprop-2-ynyl)-*N*-tosylanilines, the influence of the ancillary ligand and the arene over the cyclization products was recognized [43]. The catalyst based on the *N*-heterocyclic carbene ligand IPr [49] (IPr: 1,3-bis(2,6-diisopropyl)phenylimidazol-2-ylidene), was identified as suitable controller to favor the formation of the product arising from the migratory cyclization against that deriving from a straight iodoalkyne arylation reaction. As for the substituents on the amine ring, more electron-donating ones gave rise to the formation of the heterocycle featuring a distribution of regioisomers that indicates less iodine shift. In this context, switching from NTs to O as the linker is, intrinsically, a demanding process attempting to access 3-iodo-2*H*-chromene cores, as migration is less favorable for more electron-donating groups. So, the application of this cyclization and concomitant iodine migration strategy to synthesize the target chromenes is challenging.

On this basis, we started to investigate the feasibility of the intended synthetic approach to the target chromene scaffold exploring the reactivity of 1-chloro-4-[(3-iodoprop-2-yn-1-yl)oxy]benzene (**1a**) as model compound. For the catalyst, the gold(I) complex with the IPr ligand was systematically tested. As for the counter anion to gold, bis(trifluoromethanesulfonyl)imidate (NTf_2_) was chosen which, as early pointed out by Gagosz, renders very active catalysts [50].

An initial screening for experimental conditions showed that heating the reaction mixture at 100 °C in 1,4-dioxane provides a good result for the synthesis of the desired 6-chloro-3-iodo-2*H*-chromene (**2a**), using 3 mol % of IPrAuNTf_2_ as catalyst. Representative data concerning the selection of the solvent and the identification of convenient values for the reaction temperature and time are summarized in Table 1.

**Table 1:** Screening for conditions for the hydroarylation of 4-chlorophenyl (3-iodoprop-2-yn-1-yl) ether.

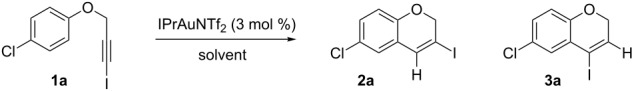					

Entry	Solvent	*T* (°C)	*t* (h)	% conversion^a^	**2a**:**3a**

1	ClCH_2_CH_2_Cl	rt	14	70	2:1
2	Et_2_O	rt	24	34	4.3:1
3	Et_2_O	40	24	62	4.5:1
4	*t*-BuOMe	56	24	–	–
5	dioxane	rt	24	40	5.2:1
6	dioxane	40	24	68	5.3:1
7	dioxane	100	2.25	98	5:1
8	CH_3_NO_2_	rt	24	–	–
9	CH_3_NO_2_	40	24	71	2.3:1
10	DMSO	80	24	–	–
11	DMF	80	24	–	–
12	ClCH_2_CH_2_Cl/CH_3_CN (1:1)	80	24	–	–

^a^Conversion determined by NMR spectroscopy using 1,3,5-trimethoxybenzene as internal standard.

For the solvent, weakly coordinating polar ethers offer a fair balance for conversion and regioselectivity. In this regard, reaction in 1,4-dioxane at 100 ºC were identified as the best experimental conditions to approach the cyclization leading to the desired 3-iodochromene **2a**. Thus, the conditions outlined in Table 1 entry 7 were chosen to broach the potential of this 1,2-iodine migration–hydroarylation process using different iodinated propargyl aryl ethers. The results are summarized in Table 2.

**Table 2:** Synthesis of 3-iodo-2*H*-chromenes.

Entry	**1**	*t* (h)	Yield (%)^a^	**2**:**3**^b^	**2**

1	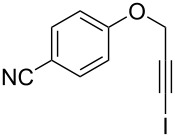 **1b**	3	91	14:1	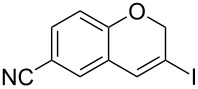 **2b**
2	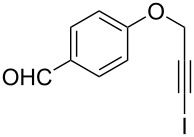 **1c**	3	87	11:1	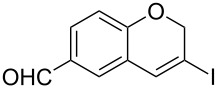 **2c**
3	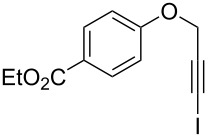 **1d**	3	97	6:1	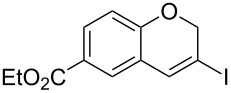 **2d**
4	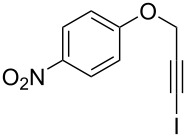 **1e**	5	88	17:1	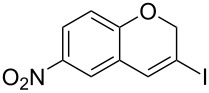 **2e**
5	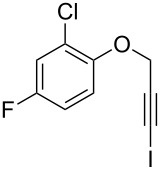 **1f**	5	90	1:0	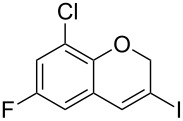 **2f**
6	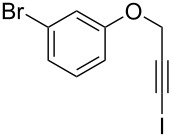 **1g**	3	96	8:1	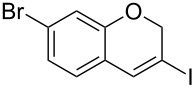 **2g** (*p*:*o* = 3:1)
7	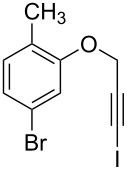 **1h**	3	89	5:1	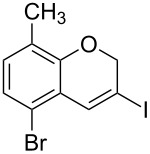 **2h**
8	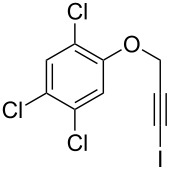 **1i**	3	93	1:0	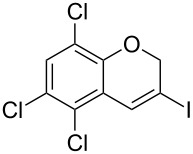 **2i**

^a^Isolated yield (mixture of regioisomers). ^b^Determined from the crude of reaction.

**Figure 1:**
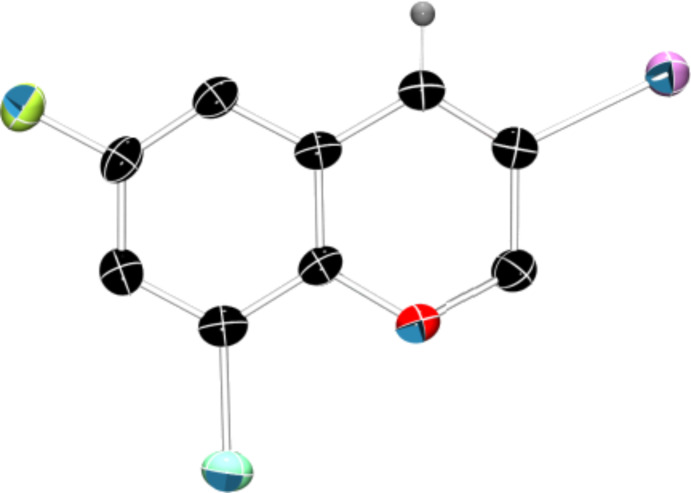
X-ray molecular structure of **2f**.

Interestingly, as no further additives are required, heating the corresponding aryl propargyl ether in dioxane under the sole influence of a relatively low catalyst loading furnishes, consistently, a significant variety of differently substituted chromenes. The selectivity in favor of the 3-iodo-substituted chromene is in all cases of practical significance. In some cases, exclusive formation of the desired 3-iodo-2*H*-chromene is noticed upon inspection of the crude reaction mixture; see, for instance, Table 2 entries 5 and 8. The structure of the prepared compounds **2** was established from their characterization data (see Supporting Information File 1). The recorded data nicely endorse the assigned structure, which was further corroborated by an X-ray analysis of **2f** (Figure 1).

The assembled collection of 4-unsubstituted iodinated heterocycles is relevant, both in terms of functional group tolerance and also for the purpose of further molecular diversification.

**Scheme 2:**
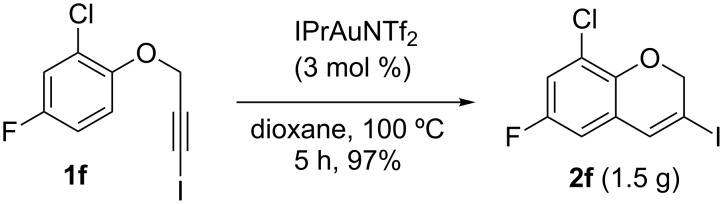
Gram-scale synthesis of **2f**.

A gram-scale reaction was conducted on the multi-halogen-containing precursor **1f**. The process is robust and 8-chloro-6-fluoro-3-iodo-2*H*-chromene (**2f**) was readily obtained (1.5 g, 97% yield) after purification by column chromatography from the reaction of 5 mmol of **1f** (1.55 g) (Scheme 2).

**Scheme 3:**
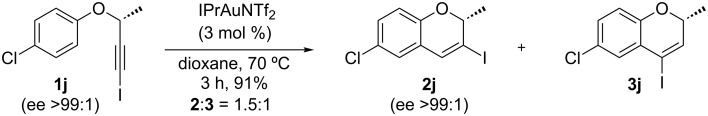
Retaining propargylic chirality.

Also, another practical issue that was addressed was the proof of the configurational stability of a chiral center in the vicinity of the alkyne. For this purpose, optically active **1j** was prepared. As next step, its reactivity in the gold(I)-catalyzed hydroarylation study was investigated (Scheme 3).

Though the regioselectivity was lower and requires further optimization, this experiment nicely reveals that chirality installed at the propargylic position in the starting material can be successfully delivered to the cyclization product, as the result of this hydroarylation with concomitant 1,2-iodine shift process.

**Scheme 4:**
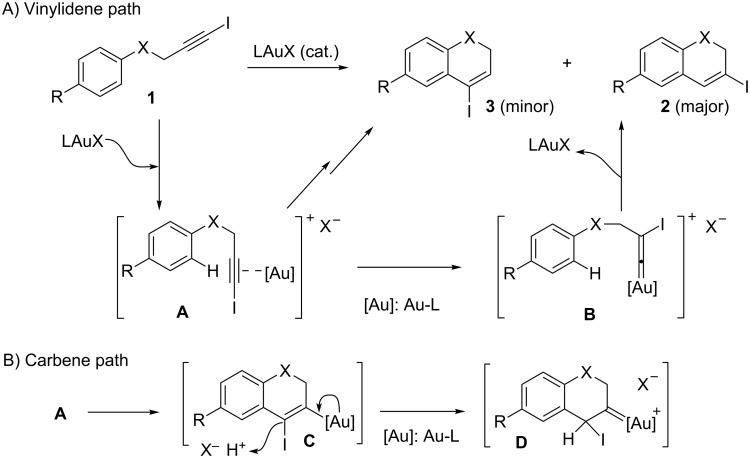
Mechanistic basis postulated for the synthesis of **2**.

Although this work deals mainly with preparative aspects for the title compounds, a preliminary mechanistic proposal to justify the obtained results could reasonably involve the generation of gold–vinylidene **B** as key intermediate behind the formation of the corresponding 3-iodo-2*H*-chromenes **2** (X = O, Scheme 4A).

Gold–vinylidenes have been proposed to mediate related hydroarylation reactions affording halogenated phenanthrenes [45] and 3-iodo-*N*-tosyl-1,2-dihydroquinoline derivatives [43]. Recent studies have provided strong evidence for their existence, and have demonstrated their powerful reactivity [51–55], identifying them as key players in ongoing activity developing the exciting notion of dual activation using gold catalysts [56].

As outlined in Scheme 4A, after an initial π-activation of the iodoalkyne, gold might trigger the generation of the β-iodo-substituted vinylidene **B**, in a process that might take place in competition with a direct Friedel–Crafts-type cyclization reaction yielding **3**. In previous work dealing with the cyclization of *N*-(3-iodoprop-2-ynyl)-*N*-tosylanilines to give related 1,2-dihydroquinolines (Scheme 4A, X = NTs) [43], we documented for a phosphite-based gold-catalyst, which render a more electrophilic metal center, an increase of the relative amount of the cyclization product **3** (4-iodo-substituted, X = NTs) at the expenses of the formation of the one with concomitant iodine shift, product **2** (3-iodo-substituted, X = NTs). On the contrary, under related conditions, a gold catalyst based on the electron-rich and neutral donor IPr ligand favors the latter cyclization against the former.

On this ground, a change in the tethering element switching the linker from NTs to oxygen (Scheme 4A, X = O) results in more activated rings towards aromatic electrophilic substitution processes. To this respect, two facts are of mechanistic significance. First, the **2**:**3** ratio for a given R substituent (Scheme 4A, R = 4-Cl) can be compared for the two linkers. For the nitrogen-containing tether (X = NTs, Scheme 4A), almost exclusive formation of **2** was noticed (reaction in 1,2-dichloroethane, at rt for 24 h, [43]). However, for the case of X = O, the corresponding value for the **2**:**3** ratio is 5:1 (Table 1, entry 7). Next, as depicted in Table 2, the herein reported cyclization takes place satisfactorily when additional electron-withdrawing groups are attached to the aromatic ring, the yield typically ranging on or above the nineties. At the same time, the selectivity of the process is dependent on the nature of the substituent R in Scheme 4A. Remarkably, the more electron-withdrawing group (R = NO_2_, Table 2, entry 4) gives similar yield and higher **2**:**3** ratio than the aldehyde (R = CHO_,_ Table 2 entry 2). For less electron-withdrawing substituents such as halogens an increase in selectivity was noticed as function of their number and nature. This is shown for the cyclization of **1a** (Table 1, entry 7) in comparison with the cyclization of **1f** and **1i** (Table 2, entries 5 and 8). These results nicely match the proposed process competition scenario. The noticed scope endorses an active involvement for a highly reactive gold–vinylidene intermediate as responsible for the selectivity of the eventual cyclization step, in agreement with the tentative mechanistic rationale depicted in Scheme 4A.

Though the substitution pattern is not the one commonly associated with conventional electrophilic aromatic substitution reactions, other mechanism should not be disregarded on the basis of the structure of the final product. So, the alternative mechanistic description summarized in Scheme 4B cannot be firmly rejected, at the moment. In this case, a demanding electrophilic substitution must occur and should produce very efficiently the 3-aurated-4-iodo-2*H*-chromene **C** and one equivalent of acid. Next, gold-assisted protonation at C-4 should afford **D** [57], an intermediate featuring a gold-carbene at C-3, that would require a subsequent and selective 1,2-iodine shift to furnish compounds **2** and regenerate the catalyst.

In this context, on the basis of the information gathered so far, and taking into account the precedents in the literature, we favor the mechanism outlined in Scheme 4A as the most likely one.

## Conclusion

In short, the reported gold-catalyzed cyclization opens up a versatile approach to the synthesis of elusive 4-unsubstituted-3-iodo-2*H*-chromenes. This transformation uses common starting materials. The resulting protocol is compatible with a significant variety of functional groups and can be easily conducted on a gram-scale.

## Experimental

All the reactions were carried out using oven dried glassware under nitrogen (99,99%) or argon (99,999%) atmosphere. Dioxane was distilled before used from sodium. Flash chromatography was performed on silica gel 60 (230–400) mesh. The solvents used in flash chromatography, hexane and ethyl acetate, were obtained from commercial suppliers and used without further purification. Cyclization reactions were performed in a RR98030 12 place Carousel Reaction Station^TM^ from Radleys Discovery Technologies, equipped with gas-tight threaded caps with a valve, cooling reflux head system, and digital temperature controller. All common reagents and solvents were obtained from commercial suppliers and used without any further purification unless otherwise noted. ^1^H NMR (300, 400 MHz) and ^13^C NMR (75.5, 100 MHz) spectra were measured in CDCl_3_, CD_2_Cl_2_ or DMSO at room temperature on a Bruker DPX-300, or Bruker AVANCE-300 MHz and 400 MHz instruments, with CHCl_3_ (δ = 7.26, ^1^H NMR; δ = 77.16, ^13^C NMR), CH_2_Cl_2_ (δ = 5.33, ^1^H NMR; δ = 54.84, ^13^C NMR) or DMSO (δ = 2.50, ^1^H NMR; δ = 39.52, ^13^C NMR) as internal standards. Data are reported as follows: chemical shift, multiplicity (s: singlet, bs: broad singlet, d: doublet, t: triplet, q: quartet, m: multiplet), coupling constants (*J* in Hz) and integration. Carbon multiplicities were assigned by DEPT and HSQC techniques. Melting points (mp) were measured on a Büchi–Totoli apparatus and are uncorrected.

### Synthesis of starting materials

Starting materials **1** were obtained from the corresponding phenols through a two steps synthetic route.

**General procedure for the propargylation of phenols**


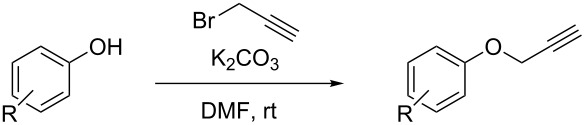


To a suspension or solution of the corresponding phenol (1 equiv; 5 mmol) in DMF (20 mL), potassium carbonate was added (2 equiv; 10 mmol) followed by a solution of propargyl bromide (commercial source: 80% in toluene) (1.5 equiv; 7.5 mmol). The reaction was controlled by TLC and when it was finished, it was diluted with Et_2_O (30 mL) and then brine was added. The organic layer was washed in a separation funnel with brine to extract all the DMF (5 times, 15 mL), dried over Na_2_SO_4_, filtered and evaporated to afford the corresponding crude mixture which, in most cases, was pure enough to use in the next step without further purification.

**Iodination of terminal alkynes**


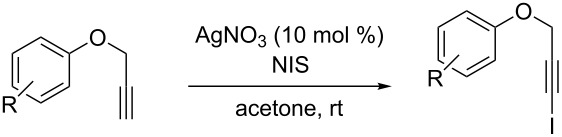


The starting alkyne (1 equiv; 2 mmol) was dissolved in acetone (10 mL). Then, silver nitrate (0.1 equiv; 0.2 mmol) and *N*-iodosuccinimide (NIS) (1.15 equiv; 2.30 mmol) were added successively. After three hours, the reaction mixture was cooled to 0 °C and filtered. The resulting crude was subjected to flash chromatography to obtain compounds **1** substantially pure.

**Preparation of ethers from phenols and chiral non-racemic propargylic alcohols**


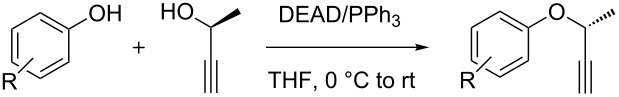


(*S*)-(−)-3-butyn-2-ol (5 mmol; commercially available, 464007 Sigma-Aldrich) was dissolved in THF (25 mL) in a flame dried round bottom flask, under nitrogen atmosphere, and the corresponding phenol (1.05 equiv, 5.25 mmol) and triphenylphosphine (1.1 equiv, 5.5 mmol) are added successively. The solution was cooled to 0 °C and diethyl azodicarboxylate (1.2 equiv, 6 mmol) was added dropwise. The ice bath was removed and the reaction was stirred overnight. The solvents were removed under reduced pressure and the resulting crude was subjected to flash chromatography to give substantially pure and optically active terminal alkynes with (*R*)-configuration.

**Cycloisomerization to give 3-iodo-2*H*-chromenes**


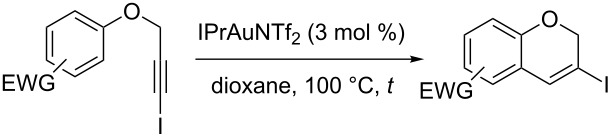


To a solution of the corresponding starting material **1** (1 equiv; 0.3 mmol) in dioxane (2 mL), under argon atmosphere, IPrAuNTf_2_ was added (0.03 equiv; 0.009 mmol) and the reaction mixture was heated at 100 °C. The reaction progress was monitored by TLC and, upon completion, solvents were removed under vacuum and the resulting crude was subjected to flash chromatography to afford the products (see specific conditions for each substrate).

**Scaled-up cycloisomerization of 2-chloro-4-fluoro-1-[(3-iodoprop-2-yn-1-yl)oxy]benzene (1f)**


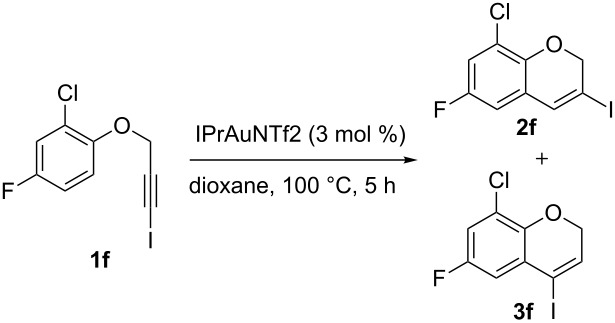


5 mmol of **1f** (1.55 g) were disposed in a flame-dried 250 mL Schlenk flask under argon and dissolved with 35 mL of dry dioxane. After complete solution of the starting material, 0.15 mmol of the catalyst (3 mol %; 0.130 g) were added and the reaction was heated at 100 °C. After 5 h, when the reaction was finished, solvents were removed in vacuum and the solid residue was purified by flash chromatography using *n*-hexane as eluent furnishing **2f** with >99:1 regioselectivity (**2:3**) and in 97% yield (1.50 g).

## Supporting Information

File 1 Characterization data for compounds **1a**–**j** and **2a**–**j**; ^1^H and ^13^C NMR spectra for compounds **1a**–**j** and **2a**–**j**; X-ray molecular structure for **2f**; HPLC chromatograms for **1j** and **2j** and structural assignment for compounds **3**.
