# Supplementary material for: Gold(I)-catalyzed hydroarylation reaction of aryl (3-iodoprop-2-yn-1-yl) ethers: synthesis of 3-iodo-2H-chromene derivatives
Source: Beilstein J Org Chem. 2013 Oct 16;9:2120–8. doi: 10.3762/bjoc.9.249 (PMC3817509; doi:10.3762/bjoc.9.249)
Supplement: File 1 — Characterization data for compounds 1a–j and 2a–j; 1H and 13C NMR spectra for compounds 1a–j and 2a–j; X-ray molecular structure for 2f; HPLC chromatograms for 1j and 2j and structural assignment for compounds 3. [file Beilstein_J_Org_Chem-09-2120-s001.pdf]

Supporting Information

for

Gold(I)-catalyzed hydroarylation reaction of aryl (3-iodoprop-2-yn-1-yl) ethers: synthesis of 3-iodo-2*H*-chromene derivatives

Pablo Morán-Poladura, Eduardo Rubio and José M. González*

Address: Departamento de Química Orgánica e Inorgánica and Instituto Universitario de Química Organometálica "Enrique Moles", Universidad de Oviedo, C/Julián Clavería 8, Oviedo, 33006, Spain

Email: José M. González* - jmgd@uniovi.es

*Corresponding author

Characterization data for compounds 1a–j and 2a–j; ^{1}H and ^{13}C NMR spectra for compounds 1a–j and 2a–j; X-ray molecular structure for 2f; HPLC chromatograms for 1j and 2j and structural assignment for compounds 3

Summary

1.- *Characterization data for compounds* **1a**–**1j** and **2a**–**2j**………………………. S2–S5

2.- *^{1}H and ^{13}C NMR spectra for compounds* **1a**–**1j** and **2a**–**2j**…………………..S6–S25

3.- *X-ray molecular structure for **2f***…………………………………………………. S26

4.- *HPLC chromatograms for **1j** and **2j***…………………………………………..S27–S28

5.- *Structural assignment for compounds **3***……………………………………...S29–S30

1.- Characterization data for compounds **1a**–**1j** and **2a**–**2j**

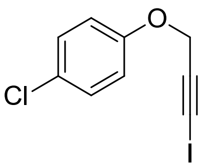

1-Chloro-4-[(3-iodoprop-2-yn-1-yl)oxy]benzene (1a)
White solid; mp 50-52 °C (lit.: 52-53 ºC [1]); Molecular formula: C_9H_6OClI; Purified by flash chromatography (Hex); HRMS (EI): calcd. for C_9H_6OClI: 291.9152, found: 291.9158; ^{1}H NMR (300 MHz, $CDCl_3$), δ: 7.28 (d, *J* = 9.1 Hz, 2H), 6.92 (d, *J* = 9.1 Hz, 2H), 4.82 (s, 2H); ^{13}C NMR (75 MHz, $CDCl_3$), δ: 156.1 (C), 129.4 (CH), 126.6 (C), 116.2 (CH), 88.7 (C), 57.6 (CH_2), 5.3 (C).

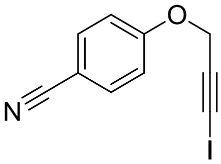

4-[(3-Iodoprop-2-yn-1-yl)oxy]benzonitrile (1b)
White solid; mp 160-161 °C (lit.: 161-162 ºC [1]); Molecular formula: $C_{10}H_6NOI$; Purified by flash chromatography (Hex:AcOEt, 20:1); HRMS (EI): calcd. for $C_{10}H_6NOI$: 282.9494, found: 282.9496; ^{1}H NMR (300 MHz, $CDCl_3$), δ: 7.63 (d, *J* = 9.0 Hz, 2H), 7.04 (d, *J* = 9.0 Hz, 2H), 4.90 (s, 2H); ^{13}C NMR (75 MHz, $CDCl_3$), δ: 160.7 (C), 134.0 (CH), 119.0 (C), 115.6 (CH), 105.0 (C), 87.8 (C), 57.4 (CH_2), 6.5 (C).

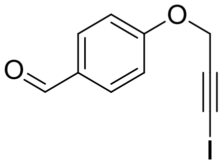

4-[(3-Iodoprop-2-yn-1-yl)oxy]benzaldehyde (1c)
White solid; mp 154-155 °C; Molecular formula: $C_{10}H_7O_2I$; Purified by flash chromatography (Hex:AcOEt, 20:1); HRMS (EI): calcd. for $C_{10}H_7O_2I$: 285.9491, found: 285.9487; ^{1}H NMR (300 MHz, $CDCl_3$), δ: 9.93 (s, 1H), 7.88 (d, *J* = 8.8 Hz, 2H), 7.09 (d, *J* = 8.8 Hz, 2H), 4.93 (s, 2H); ^{13}C NMR (75 MHz, $CDCl_3$), δ: 190.8 (CH), 162.3 (C), 131.9 (CH), 130.6 (C), 115.1 (CH), 88.1 (C), 57.4 (CH_2), 6.1 (C).

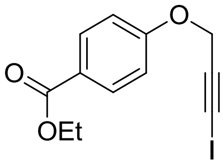

Ethyl 4-[(3-iodoprop-2-yn-1-yl)oxy]benzoate (1d)
White solid; mp 90-91 °C; Molecular formula: $C_{12}H_{11}O_3I$; Purified by flash chromatography (Hex:AcOEt, 40:1); HRMS (EI): calcd. for $C_{12}H_{11}O_3I$: 329.9753, found: 329.9756; ^{1}H NMR (300 MHz, $CDCl_3$), δ: 8.02 (d, *J* = 9.0 Hz, 2H), 6.99 (d, *J* = 9.0 Hz, 2H), 4.89 (s, 2H), 4.36 (q, *J* = 7.1 Hz, 2H), 1.39 (t, *J* = 7.1 Hz, 3H); ^{13}C NMR (75 MHz, $CDCl_3$), δ: 166.6 (C), 161.5 (C), 131.9 (CH), 124.2 (C), 114.8 (CH), 88.8 (C), 61.1 (CH_2), 57.7 (CH_2), 14.8 (CH_3), 6.1 (C).

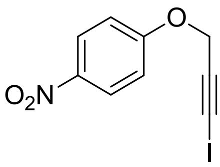

1-[(3-Iodoprop-2-yn-1-yl)oxy]-4-nitrobenzene (1e)
Yellow solid; decomp.: 187-189 °C (lit.: 184-185 ºC [1]); Molecular formula: $C_9H_6NO_3I$; Purified by flash chromatography (Hex:AcOEt, 10:1); HRMS (EI): calcd. for $C_9H_6NO_3I$: 302.9392, found: 302.9395; ^{1}H NMR

(300 MHz, DMSO-d_6), δ: 8.23 (d, *J* = 9.3 Hz, 2H), 7.18 (d, *J* = 9.3 Hz, 2H), 5.10 (s, 2H); ^{13}C NMR (75 MHz, DMSO-d_6), δ: 162.8 (C), 141.8 (C), 126.3 (CH), 115.8 (CH), 87.8 (C), 58.3 (CH_2), 16.6 (C).

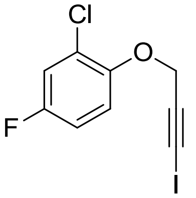

2-Chloro-4-fluoro-1-[(3-iodoprop-2-yn-1-yl)oxy]benzene (1f)
Pale yellow solid; mp 45-46 °C; Molecular formula: C_9H_5OFClI; Purified by flash chromatography (Hex); HRMS (EI): calcd. for C_9H_5OFClI: 309.9058, found: 309.9057; ^{1}H NMR (300 MHz, $CDCl_3$), δ: 7.16 (dd, *J* = 8.0, 2.9 Hz, 1H), 7.06 (dd, *J* = 9.1, 4.9 Hz, 1H), 6.97 (ddd, *J* = 9.1, 7.7, 2.9 Hz, 1H), 4.90 (s, 2H); ^{13}C NMR (75 MHz, $CDCl_3$), δ: 157.3 (d, *J* = 243.6 Hz, C), 149.7 (d, *J* = 2.3 Hz, C), 124.3 (d, *J* = 10.6 Hz, C), 117.8 (d, *J* = 26.1 Hz, CH), 115.9 (d, *J* = 8.7 Hz, CH), 114.1 (d, *J* = 22.7 Hz, CH), 88.5 (s, C), 59.1 (s, CH_2), 6.0 (s, C); ^{19}F NMR (282 MHz, $CDCl_3$), δ: -119.8.

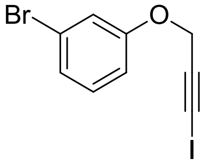

1-Bromo-3-[(3-iodoprop-2-yn-1-yl)oxy]benzene (1g)
Colourless oil; Molecular formula: C_9H_6OBrI; Purified by flash chromatography (Hex); HRMS (EI): calcd. for C_9H_6OBrI: 335.8647, found: 335.8649; ^{1}H NMR (300 MHz, $CDCl_3$), δ: 7.23-7.12 (m, 3H), 6.92 (d, *J* = 7.5 Hz, 1H), 4.83 (s, 2H); ^{13}C NMR (75 MHz, $CDCl_3$), δ: 158.2 (C), 130.6 (CH), 124.8 (CH), 122.8 (C), 118.4 (CH), 113.7 (CH), 88.5 (C), 57.5 (CH_2), 5.6 (C).

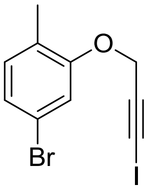

4-Bromo-2-[(3-iodoprop-2-yn-1-yl)oxy]-1-methylbenzene (1h)
White solid; mp 77-78 °C; Molecular formula: $C_{10}H_8OBrI$; Purified by flash chromatography (Hex); HRMS (EI): calcd. for $C_{10}H_8OBrI$: 349.8803, found: 349.8802; ^{1}H NMR (300 MHz, CD_2Cl_2), δ: 7.12-7.03 (m, 3H), 4.88 (s, 2H), 2.20 (s, 3H); ^{13}C NMR (75 MHz, CD_2Cl_2), δ: 156.8 (C), 132.3 (CH), 126.8 (C), 124.6 (CH), 119.6 (C), 115.5 (CH), 89.3 (C), 58.1 (CH_2), 16.0 (CH_3), 5.1 (C).

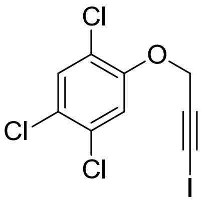

1,2,4-Trichloro-5-[(3-iodoprop-2-yn-1-yl)oxy]benzene (1i)
White solid; mp 114-115 °C (lit.: 114-115 ºC [1]); Molecular formula: $C_9H_4OCl_3I$; Purified by flash chromatography (Hex); HRMS (EI): calcd. for $C_9H_4OCl_3I$: 359.8372, found: 359.8374; ^{1}H NMR (300 MHz, $CDCl_3$), δ: 7.49 (s, 1H), 7.16 (s, 1H), 4.91 (s, 2H); ^{13}C NMR (75 MHz, $CDCl_3$), δ: 152.5 (C), 131.6 (C), 131.5 (CH), 125.8 (C), 123.0 (C), 116.3 (CH), 87.9 (C), 59.2 (CH_2), 7.5 (C).

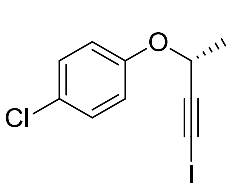

(*R*)-1-Chloro-4-[(4-iodobut-3-yn-2-yl)oxy]benzene (1j)
White solid; mp 79-80 °C; Molecular formula: $C_{10}H_8OClI$; Purified by flash chromatography (Hex); HRMS (EI): calcd. for $C_{10}H_8OClI$: 305.9308, found: 305.9300; Specific rotation (T =25,8 °C; c =0,0100 g/mL in CH_2Cl_2), $[\alpha]_D$= 201,40 deg·cm^3/g·dm ; ^{1}H NMR (300 MHz, $CDCl_3$), δ: 7.27 (d, *J* = 9.0 Hz, 2H), 6.94 (d, *J* = 9.0 Hz, 2H), 4.93 (q, *J* = 6.6 Hz, 1H), 1.67 (d, *J* = 6.6 Hz, 3H); ^{13}C NMR (75 MHz, $CDCl_3$), δ: 156.3 (C), 129.7 (CH), 126.8 (C), 117.5 (CH), 93.7 (C), 65.9 (CH), 22.7 (CH_3), 4.1 (C).

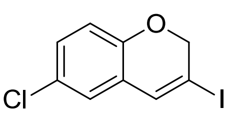

6-Chloro-3-iodo-2*H*-chromene (2a)
White solid; mp 79-80 °C; Molecular formula: C_9H_6OClI; Purified by flash chromatography (Hex); HRMS (EI): calcd. for C_9H_6OClI: 291.9152, found: 291,9153; ^{1}H NMR (401 MHz, CD_2Cl_2), δ: 7.11 (dd, *J* = 8.6, 2.5 Hz, 1H), 7.01 (bs, 1H), 6.92 (d, *J* = 2.5 Hz, 1H), 6.74 (d, *J* = 8.6 Hz, 1H), 4.90 (d, *J* = 1.7 Hz, 2H); ^{13}C NMR (75

MHz, CD_2Cl_2), δ: 150.9 (C), 132.7 (CH), 129.2 (CH), 126.2 (C), 125.2 (CH), 124.2 (C), 117.2 (CH), 89.7 (C), 73.8 (CH_2).

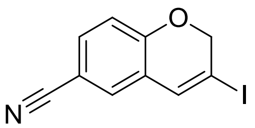

3-Iodo-2*H*-chromene-6-carbonitrile (2b)

White solid; mp 94-95 °C; Molecular formula: $C_{10}H_6NOI$; Purified by flash chromatography (Hex:AcOEt, 30:1); HRMS (EI): calcd. for $C_{10}H_6NOI$: 282.9494, found: 282.9493; ^1H NMR (300 MHz, CD_2Cl_2), δ: 7.46 (dd, J = 8.4, 2.0 Hz, 1H), 7.21 (d, J = 2.0 Hz, 1H), 7.05 (bs, 1H), 6.84 (d, J = 8.4 Hz, 1H), 5.02 (d, J = 1.8 Hz, 2H); ^{13}C NMR (75 MHz, CD_2Cl_2), δ: 156.1 (C), 134.3 (CH), 132.3 (CH), 129.8 (CH), 123.5 (C), 119.0 (C), 117.2 (CH), 105.3 (C), 90.7 (C), 74.5 (CH_2).

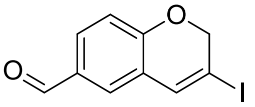

3-Iodo-2*H*-chromene-6-carbaldehyde (2c)

White solid; mp 120-121 °C; Molecular formula: $C_{10}H_7O_2I$; Purified by flash chromatography (Hex:AcOEt, 30:1); HRMS (EI): calcd. for $C_{10}H_7O_2I$: 285.9491, found: 285.9490; ^1H NMR (300 MHz, CD_2Cl_2), δ: 9.83 (s, 1H), 7.68 (dd, J = 8.3, 2.0 Hz, 1H), 7.43 (d, J = 2.0 Hz, 1H), 7.10 (bs, 1H), 6.88 (d, J = 8.3 Hz, 1H), 5.01 (d, J = 1.7 Hz, 2H); ^{13}C NMR (75 MHz, CD_2Cl_2), δ: 190.6 (CH), 157.8 (C), 133.1 (CH), 132.6 (CH), 131.1 (C), 127.3 (CH), 123.1 (C), 116.8 (CH), 89.7 (C), 74.5 (CH_2).

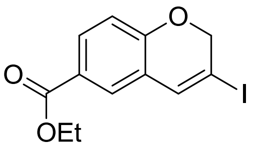

Ethyl 3-iodo-2*H*-chromene-6-carboxylate (2d)

White solid; mp 72-73 °C; Molecular formula: $C_{12}H_{11}O_3I$; Purified by flash chromatography (Hex); HRMS (EI): calcd. for $C_{12}H_{11}O_3I$: 329.9753, found: 329.9750; ^1H NMR (400 MHz, CD_2Cl_2), δ: 7.84 (dd, J = 8.5, 2.1 Hz, 1H), 7.61 (d, J = 2.1 Hz, 1H), 7.08 (bs, 1H), 6.80 (d, J = 8.5 Hz, 1H), 4.98 (d, J = 1.7 Hz, 2H), 4.33 (q, J = 7.1 Hz, 2H), 1.38 (t, J = 7.1 Hz, 3H); ^{13}C NMR (100 MHz, CD_2Cl_2), δ: 165.6 (C), 156.0 (C), 133.0 (CH), 131.4 (CH), 127.3 (CH), 124.0 (C), 122.3 (C), 115.7 (CH), 88.6 (C), 74.0 (CH_2), 60.8 (CH_2), 14.1 (CH_3).

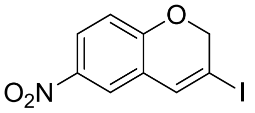

3-Iodo-6-nitro-2*H*-chromene (2e)

Yellow solid; mp 112-113 °C; Molecular formula: $C_9H_6NO_3I$; Purified by flash chromatography (Hex:AcOEt, 10:1); HRMS (EI): calcd. for $C_9H_6NO_3I$: 302.9392, found: 302.9394; ^1H NMR (300 MHz, DMSO), δ: 8.03 (dd, J = 8.8, 2.8 Hz, 1H), 7.99 (d, J = 2.7 Hz, 1H), 7.36 (bs, 1H), 6.93 (d, J = 8.8 Hz, 1H), 5.08 (d, J = 1.7 Hz, 2H); ^{13}C NMR (75 MHz, DMSO), δ: 158.2 (C), 142.2 (C), 131.8 (CH), 126.4 (CH), 123.3 (C), 122.1 (CH), 117.2 (CH), 93.5 (C), 74.6 (CH_2).

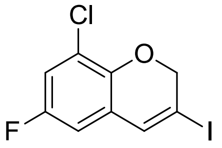

8-Chloro-6-fluoro-3-iodo-2*H*-chromene (2f)

White solid; mp 129-130 °C; Molecular formula: C_9H_5OFClI; Purified by flash chromatography (Hex); HRMS (EI): calcd. for C_9H_5OFClI: 309.9058, found: 309.9053; ^1H NMR (300 MHz, CD_2Cl_2), δ: 7.04 (bs, 1H), 6.99 (dd, J = 8.3, 2.9 Hz, 1H), 6.64 (dd, J = 8.1, 2.9 Hz, 1H), 4.99 (d, J = 1.6 Hz, 2H); ^{13}C NMR (75 MHz, CD_2Cl_2), δ: 156.8 (d, J = 242.4 Hz, C), 144.9 (d, J = 2.6 Hz, C), 132.9 (d, J = 2.0 Hz, CH), 125.0 (d, J = 9.2 Hz, C), 121.8 (d, J = 10.9 Hz, C), 117.1 (d, J = 26.3 Hz, CH), 111.2 (d, J = 24.0 Hz, CH), 91.3 (s, C), 74.6 (s, CH_2); ^{19}F NMR (282 MHz, CD_2Cl_2), δ: -121.2.

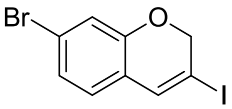

7-bromo-3-iodo-2*H*-chromene (2g)

Unseparable regioisomeric mixture of **2g**-(7-bromo-3-iodo-2*H*-chromene) and **2g´**-(5-bromo-3-iodo-2*H*-chromene) (**2g**:**2g´**, 3:1); Molecular formula: C_9H_6OBrI; Purified by flash chromatography (Hex); HRMS (EI): calcd. for C_9H_6OBrI: 335.8647, found: 335.8630; ^1H NMR (300 MHz, CD_2Cl_2), δ: 7.41 (bs, 1H-**2g´**), 7.17 (dd, J = 8.0, 1.1 Hz, 1H-**2g´**), 7.10 – 6.95 (m, 3H-**2g**, 1H-**2g´**), 6.85 – 6.75 (m, 1H-**2g**, 1H-**2g´**), 4.91 (d, J = 1.7 Hz, 2H-**2g**), 4.89 (d, J = 1.6 Hz, 2H-**2g´**); ^{13}C NMR (75

MHz, CD_2Cl_2), δ: 153.55(C-**2g´**), 152.9 (C-**2g**), 133.0 (CH-**2g**), 132.5 (CH-**2g´**), 130.1 (CH-**2g´**), 126.7 (CH-**2g**), 125.6 (CH-**2g´**), 124.6 (CH-**2g**), 122.8 (C-**2g´**), 122.1 (C-**2g**), 121.9 (C-**2g**), 120.1 (C-**2g´**), 119.1 (CH-**2g**), 115.4 (CH-**2g´**), 89.9 (C-**2g´**), 88.2 (C-**2g**), 73.8 (CH_2-**2g**), 73. 7 (CH_2-**2g´**).

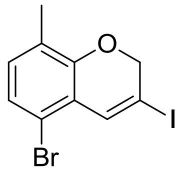

5-Bromo-3-iodo-8-methyl-2*H*-chromene (2h)
White solid; decomp.:160-162°C; Molecular formula: $C_{10}H_8OBrI$; Purified by flash chromatography (Hex); HRMS (EI) calcd. for $C_{10}H_8OBrI$: 349.8803, found: 349.8798; ^1H NMR (400 MHz), δ: 7.38 (t, J = 1.7 Hz, 1H), 7.04 (d, J = 8.1 Hz, 1H), 6.91 (d, J = 8.3 Hz, 1H), 4.89 (d, J = 1.7 Hz, 2H), 2.13 (s, 3H); ^{13}C NMR (100 MHz, CD_2Cl_2), δ: 151.5 (C), 132.8 (CH), 131.7 (CH), 125.1 (C), 124.8 (CH), 122.2 (C), 117.2 (C), 89.5 (C), 73.6 (CH_2), 15.1 (CH_3).

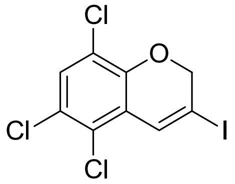

5,6,8-Trichloro-3-iodo-2*H*-chromene (2i)
White solid; m.p. =119-120°C; Molecular formula: $C_9H_4OCl_3I$; Purified by flash chromatography (Hex); HRMS (EI) calcd. for $C_9H_4OCl_3I$: 359.8372, found: 359.8370; ^1H NMR (300 MHz, CD_2Cl_2), δ: 7.46 (t, J = 1.7 Hz, 1H), 7.37 (s, 1H), 5.02 (d, J = 1.7 Hz, 3H); ^{13}C NMR (75 MHz, CD_2Cl_2), δ: 147.6 (C), 129.9 (2 x CH), 126.7 (C), 125.2 (C), 123.1 (C), 120.4 (C), 91.8 (C), 73.9 (CH_2).

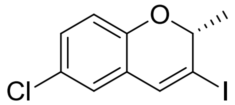

(*R*)-6-Chloro-3-iodo-2-methyl-2*H*-chromene (2j)
Pale yellow oil; Molecular formula: $C_{10}H_8OClI$; Purified by flash chromatography (Hex); HRMS (EI): calcd. for $C_{10}H_8OClI$: 305.9308, found: 305.9306; Specific rotation (T =25,8°C; c =0,0108 g/mL in CH_2Cl_2), $[\alpha]_D$= -109,50 deg·cm^3/g·dm; ^1H NMR (300 MHz, CD_2Cl_2), δ: 7.13 (dd, J = 8.6, 2.5 Hz, 1H), 6.99 (bs, 1H), 6.94 (d, J = 2.5 Hz, 1H), 6.77 (d, J = 8.7 Hz, 1H), 5.07 (qd, J = 6.6, 0.6 Hz, 1H), 1.48 (d, J = 6.6 Hz, 3H); ^{13}C NMR (75 MHz, CD_2Cl_2), δ: 150.1 (C), 132.7 (CH), 129.6 (CH), 126.3 (C), 125.4 (CH), 124.4 (C), 118.3 (CH), 96.4 (C), 79.4 (CH), 19.1 (CH_3).

References

1. Seki, S. Nomiya, B. Owaga, H. Halopropargyl aryl ethers. JP Patent 39019791, Sep 12, 1964

2. ^{1}H and ^{13}C NMR spectra for compounds **1a**–**1j** and **2a**–**2j**

1a

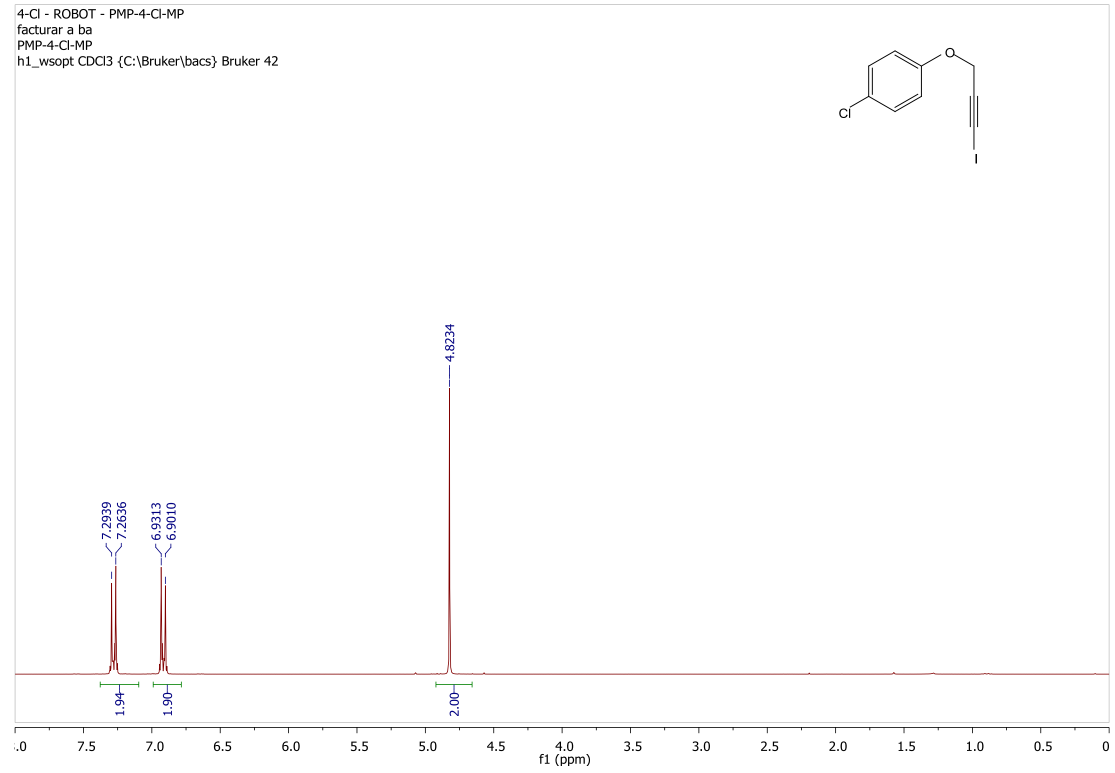

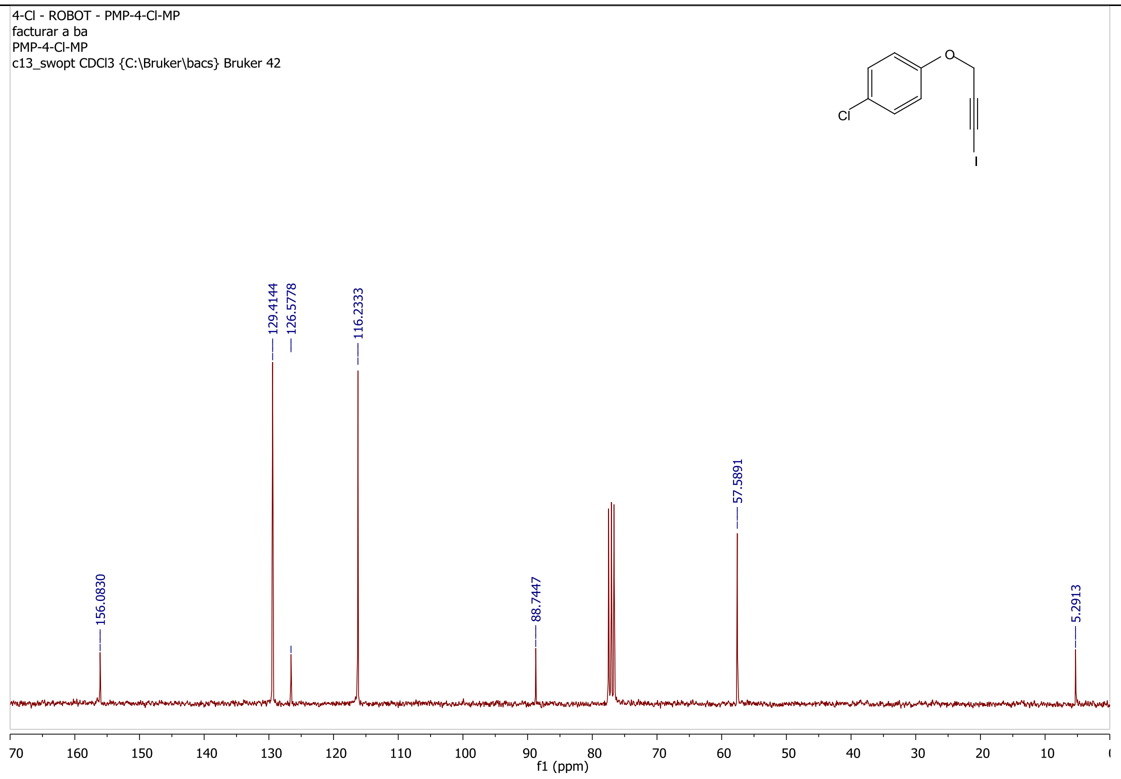

1b

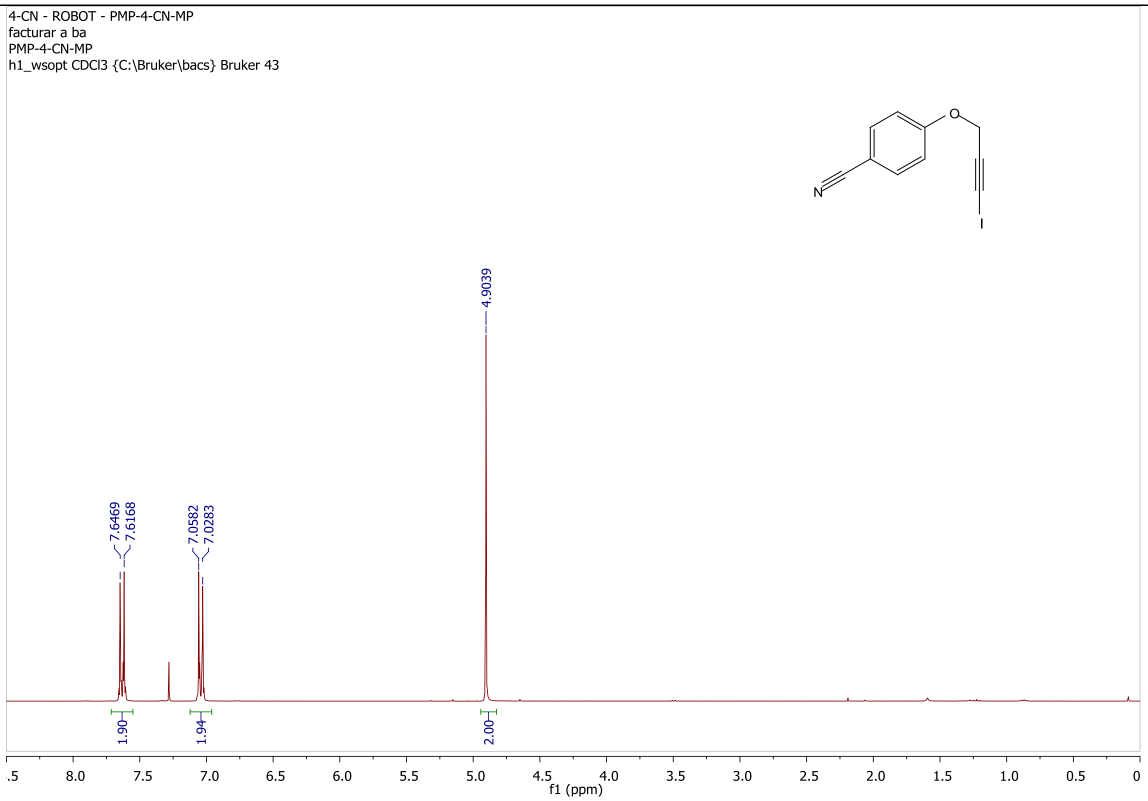
4-CN - ROBOT - PMP-4-CN-MP
facturar a ba
PMP-4-CN-MP
h1_wsopt CDCl3 {C:\Bruker\bacs} Bruker 43
N
O
I
7.6469
7.6168
7.0582
7.0283
4.9039
1.90
1.94
2.00
.5
8.0
7.5
7.0
6.5
6.0
5.5
5.0
4.5
4.0
3.5
3.0
2.5
2.0
1.5
1.0
0.5
0
f1 (ppm)

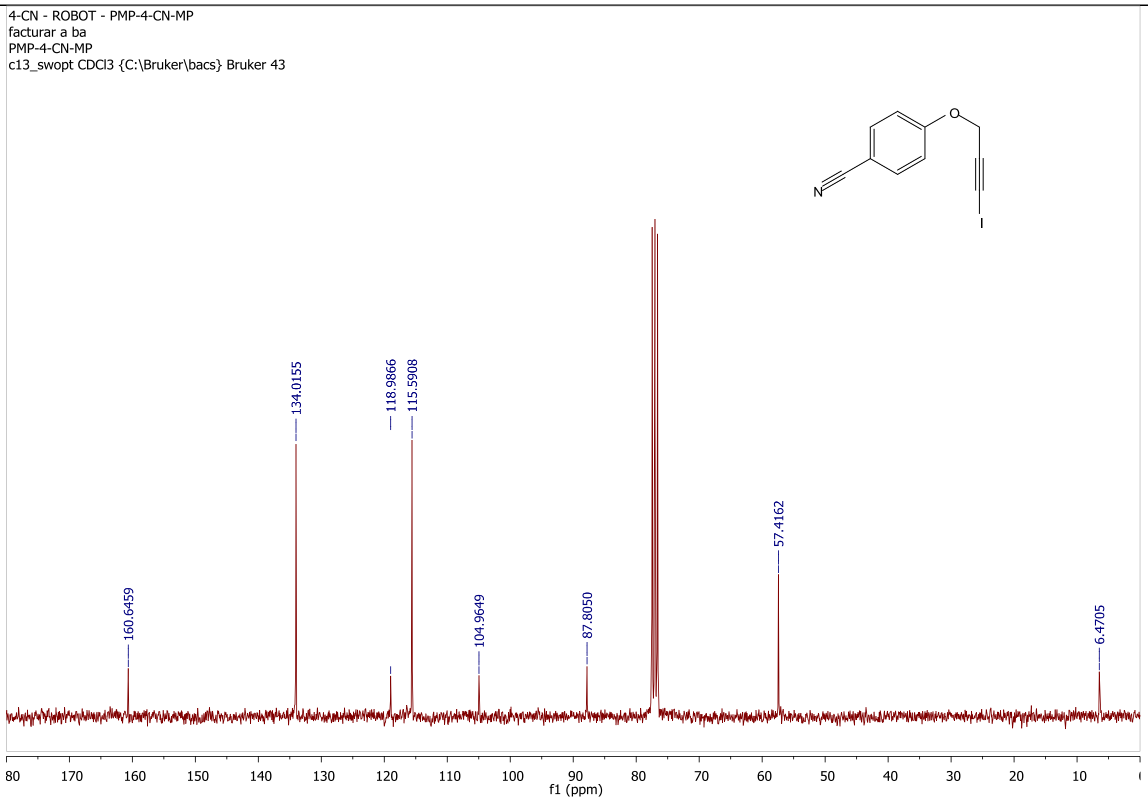
4-CN - ROBOT - PMP-4-CN-MP
facturar a ba
PMP-4-CN-MP
c13_swopt CDCl3 {C:\Bruker\bacs} Bruker 43
N
O
I
160.6459
134.0155
118.9866
115.5908
104.9649
87.8050
57.4162
6.4705
80
170
160
150
140
130
120
110
100
90
80
70
60
50
40
30
20
10
f1 (ppm)

1c

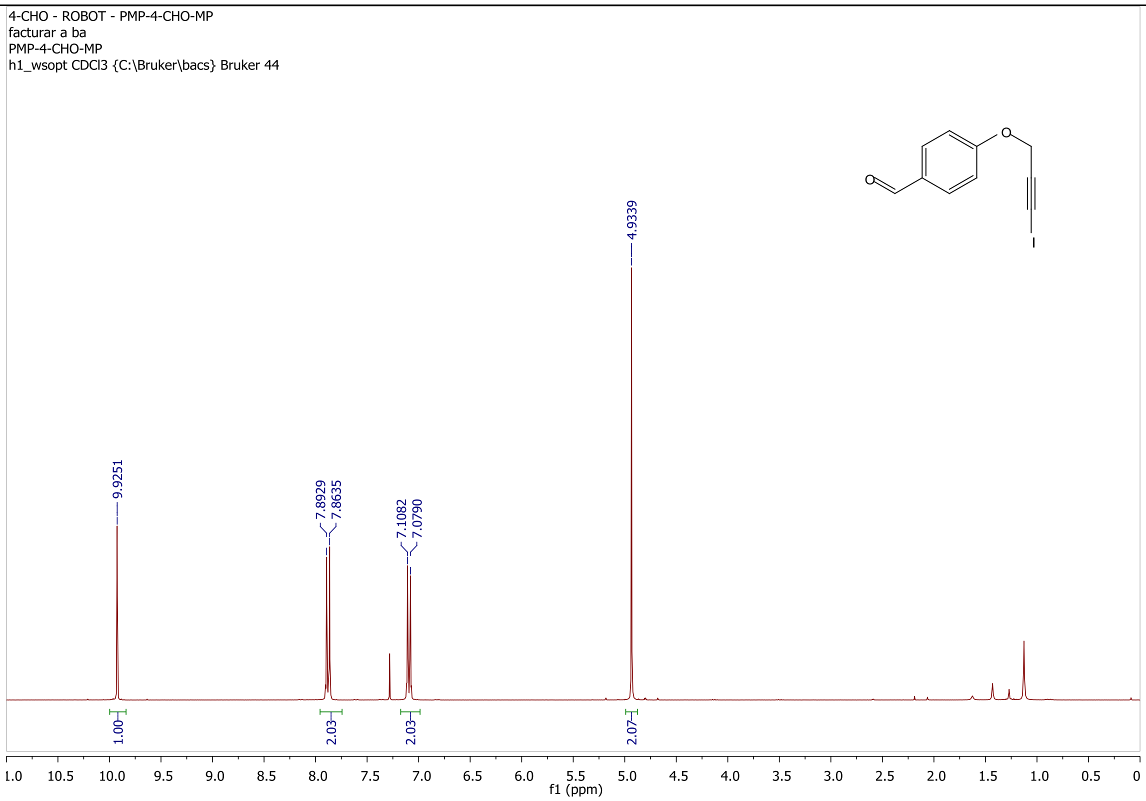
4-CHO - ROBOT - PMP-4-CHO-MP
facturar a ba
PMP-4-CHO-MP
h1_wsopt CDCl3 {C:\Bruker\bacs} Bruker 44
9.9251
7.8929
7.8635
7.1082
7.0790
4.9339
1.00
2.03
2.03
2.07
f1 (ppm)
O
I

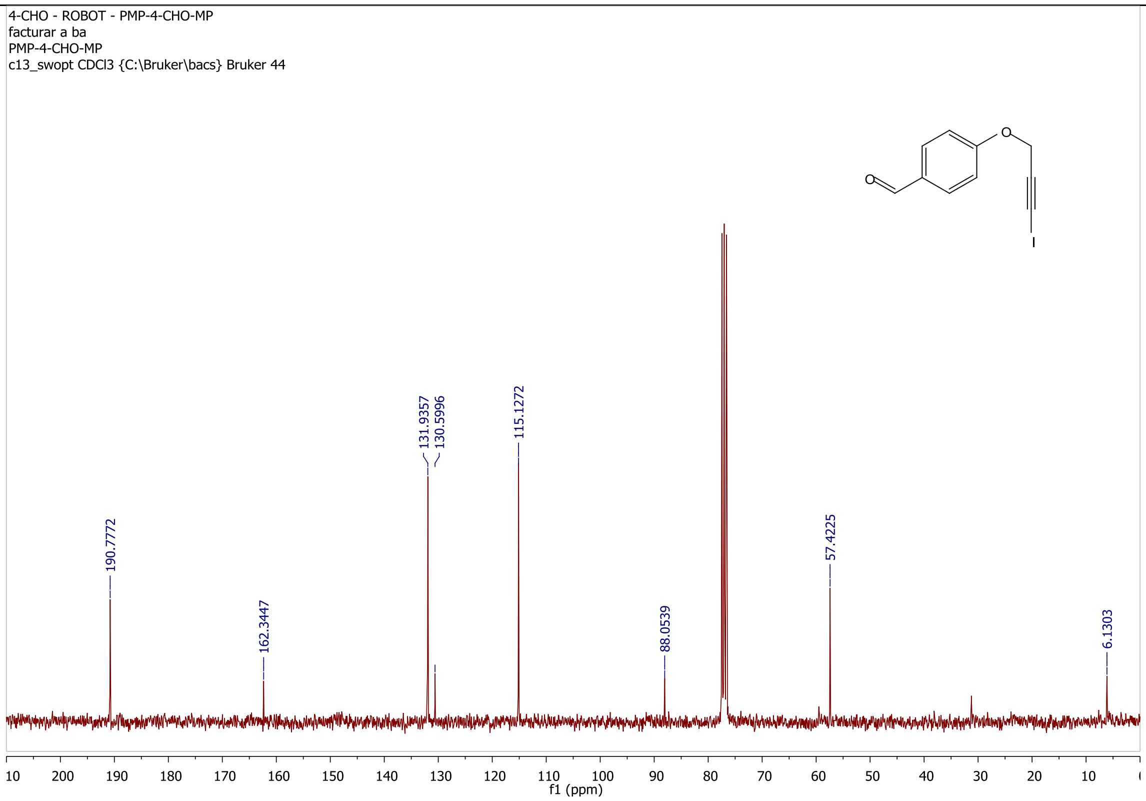
4-CHO - ROBOT - PMP-4-CHO-MP
facturar a ba
PMP-4-CHO-MP
c13_swopt CDCl3 {C:\Bruker\bacs} Bruker 44
190.7772
162.3447
131.9357
130.5996
115.1272
88.0539
57.4225
6.1303
f1 (ppm)
O
I

1d

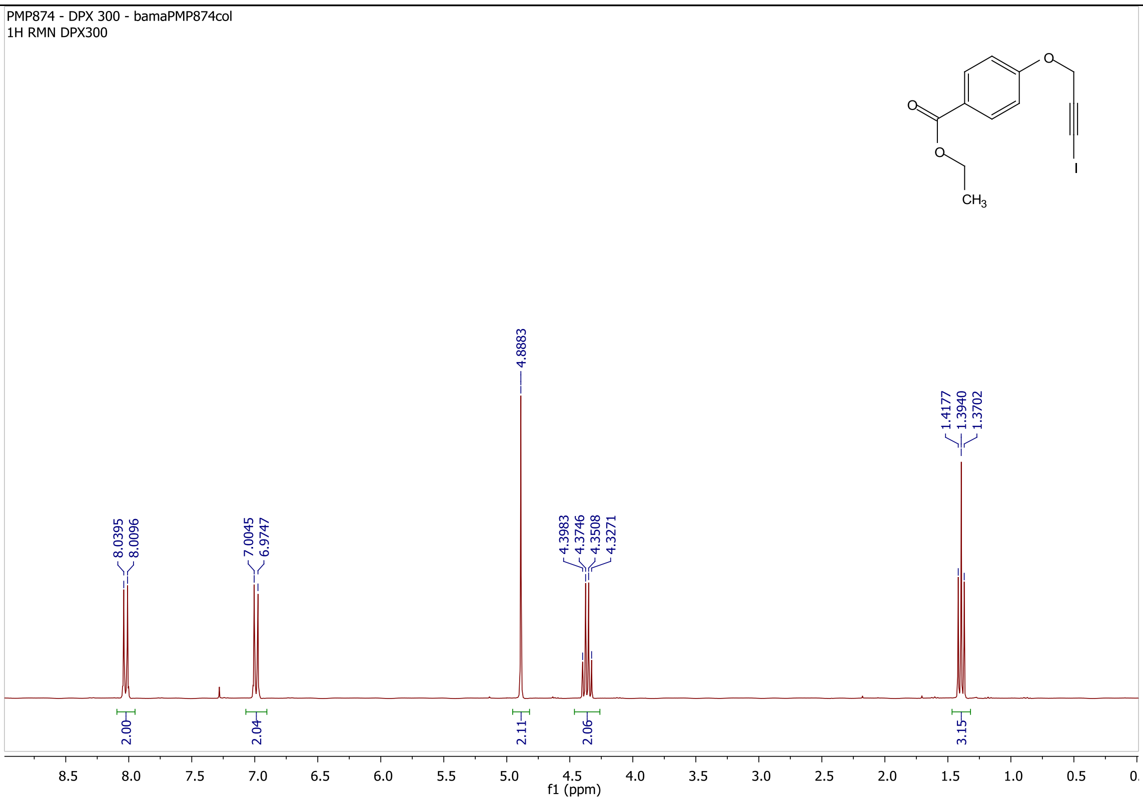
PMP874 - DPX 300 - bamaPMP874col
1H RMN DPX300
O
O
O
I
CH3
8.0395
8.0096
7.0045
6.9747
4.8883
4.3983
4.3746
4.3508
4.3271
1.4177
1.3940
1.3702
2.00
2.04
2.11
2.06
3.15
8.5 8.0 7.5 7.0 6.5 6.0 5.5 5.0 4.5 4.0 3.5 3.0 2.5 2.0 1.5 1.0 0.5
f1 (ppm)

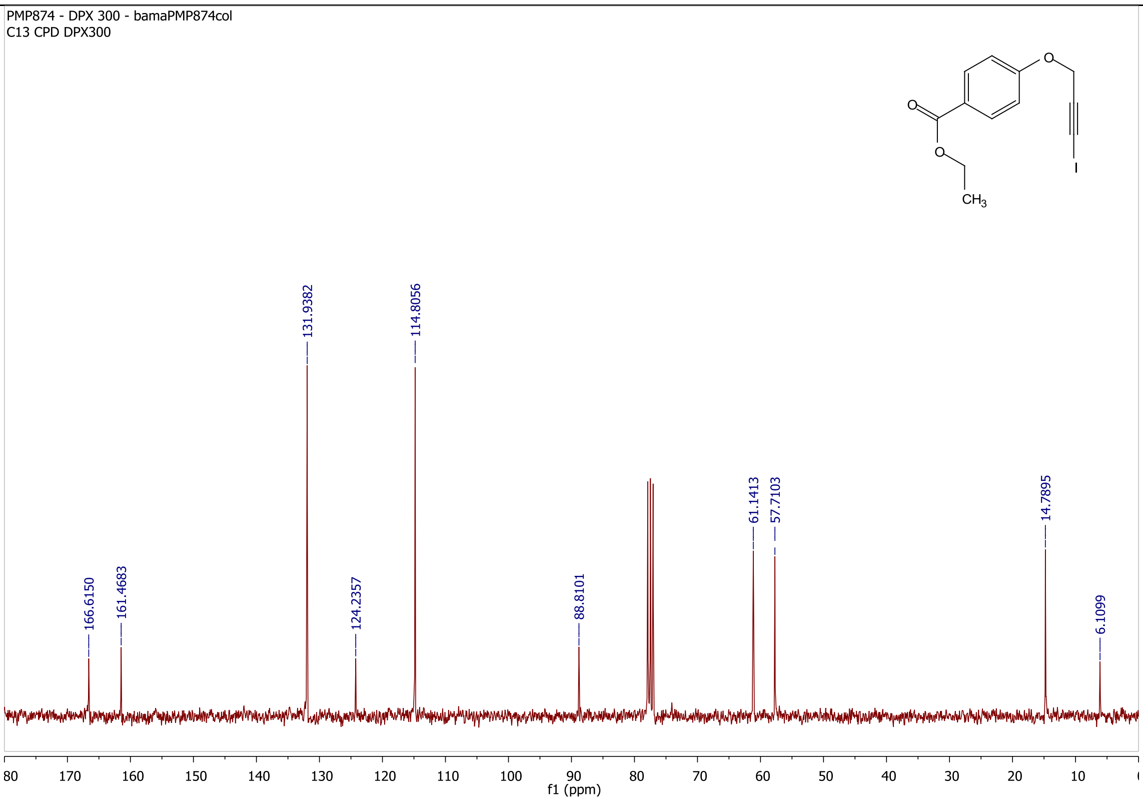
PMP874 - DPX 300 - bamaPMP874col
C13 CPD DPX300
O
O
O
I
CH3
166.6150
161.4683
131.9382
124.2357
114.8056
88.8101
61.1413
57.7103
14.7895
6.1099
170 160 150 140 130 120 110 100 90 80 70 60 50 40 30 20 10
f1 (ppm)

1e

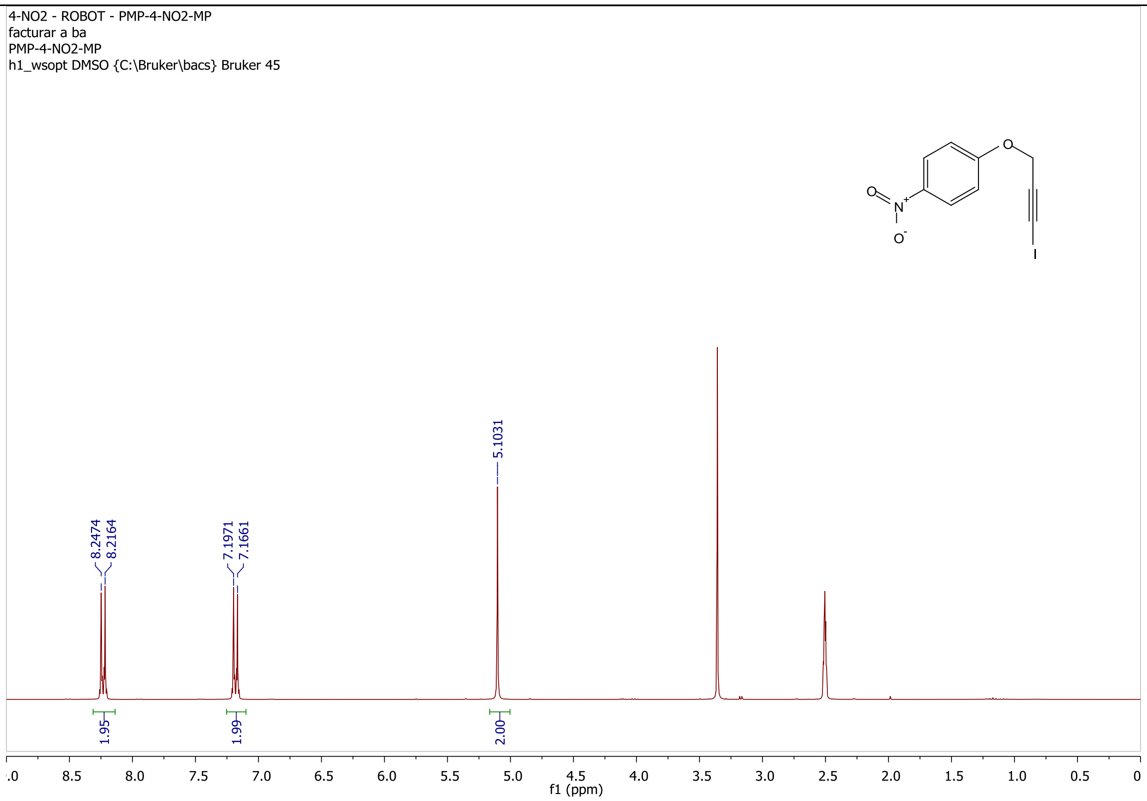

4-NO2 - ROBOT - PMP-4-NO2-MP
facturar a ba
PMP-4-NO2-MP
h1_wsopt DMSO {C:\Bruker\bacs} Bruker 45
8.2474
8.2164
7.1971
7.1661
5.1031
1.95
1.99
2.00
f1 (ppm)

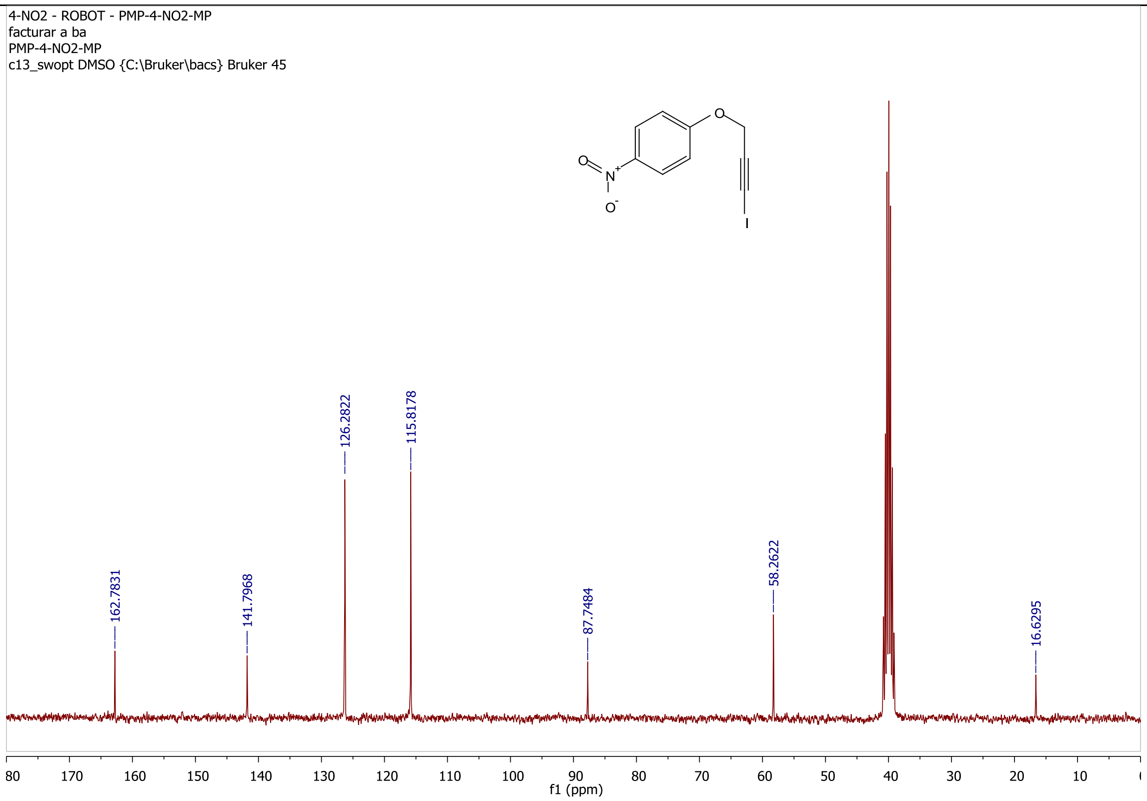

4-NO2 - ROBOT - PMP-4-NO2-MP
facturar a ba
PMP-4-NO2-MP
c13_swopt DMSO {C:\Bruker\bacs} Bruker 45
162.7831
141.7968
126.2822
115.8178
87.7484
58.2622
16.6295
f1 (ppm)

1f

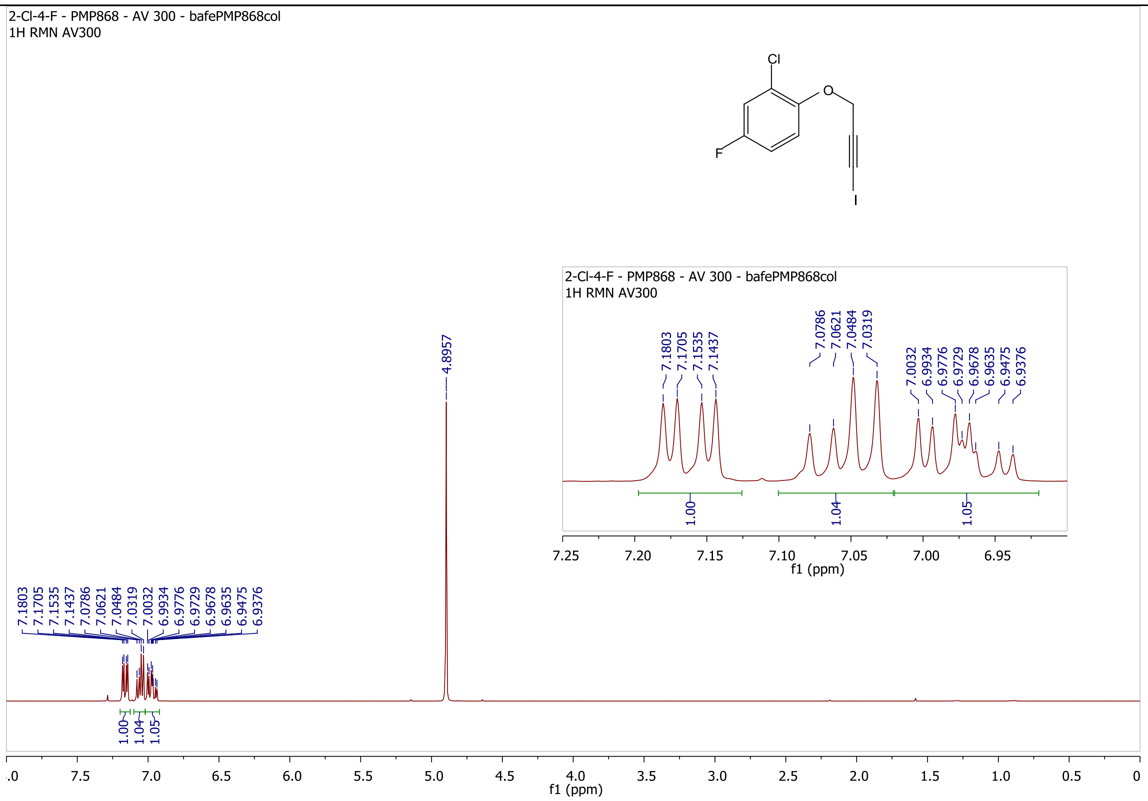
2-Cl-4-F - PMP868 - AV 300 - bafePMP868col
1H RMN AV300
Cl
O
F
I
7.1803
7.1705
7.1535
7.1437
7.0786
7.0621
7.0484
7.0319
7.0032
6.9934
6.9776
6.9729
6.9678
6.9635
6.9475
6.9376
4.8957
1.00
1.04
1.05
f1 (ppm)

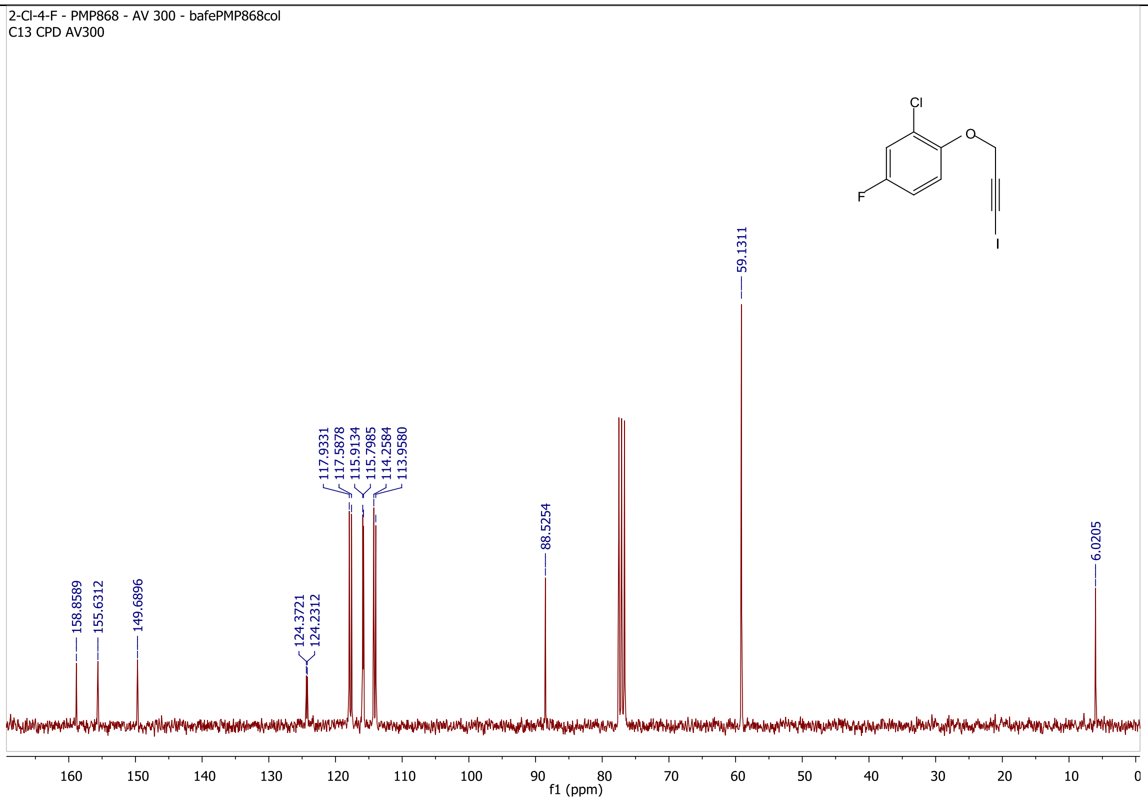
2-Cl-4-F - PMP868 - AV 300 - bafePMP868col
C13 CPD AV300
Cl
O
F
I
158.8589
155.6312
149.6896
124.3721
124.2312
117.9331
117.5878
115.9134
115.7985
114.2584
113.9580
88.5254
59.1311
6.0205
f1 (ppm)

1g

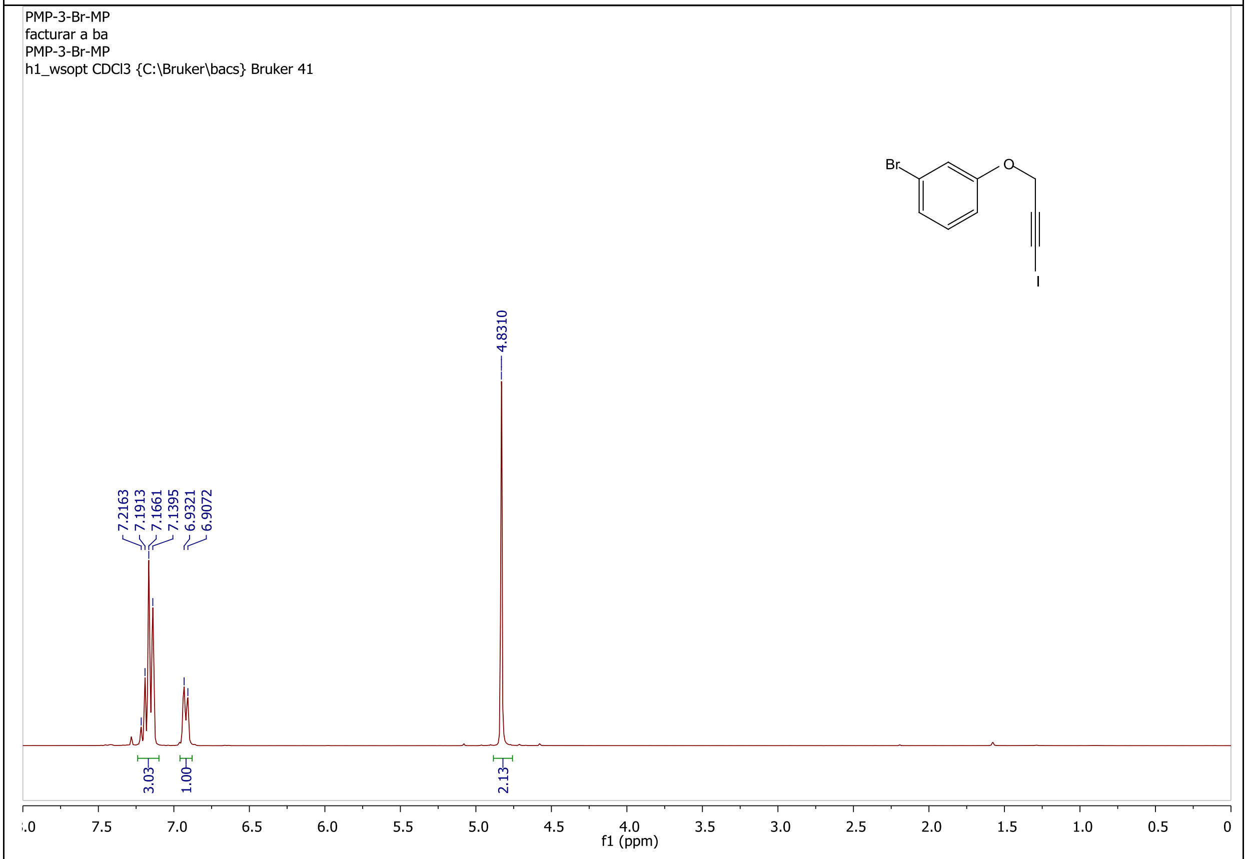
PMP-3-Br-MP
facturar a ba
PMP-3-Br-MP
h1_wsopt CDCl3 {C:\Bruker\bacs} Bruker 41
Br
O
I
7.2163
7.1913
7.1661
7.1395
6.9321
6.9072
4.8310
3.03
1.00
2.13
f1 (ppm)

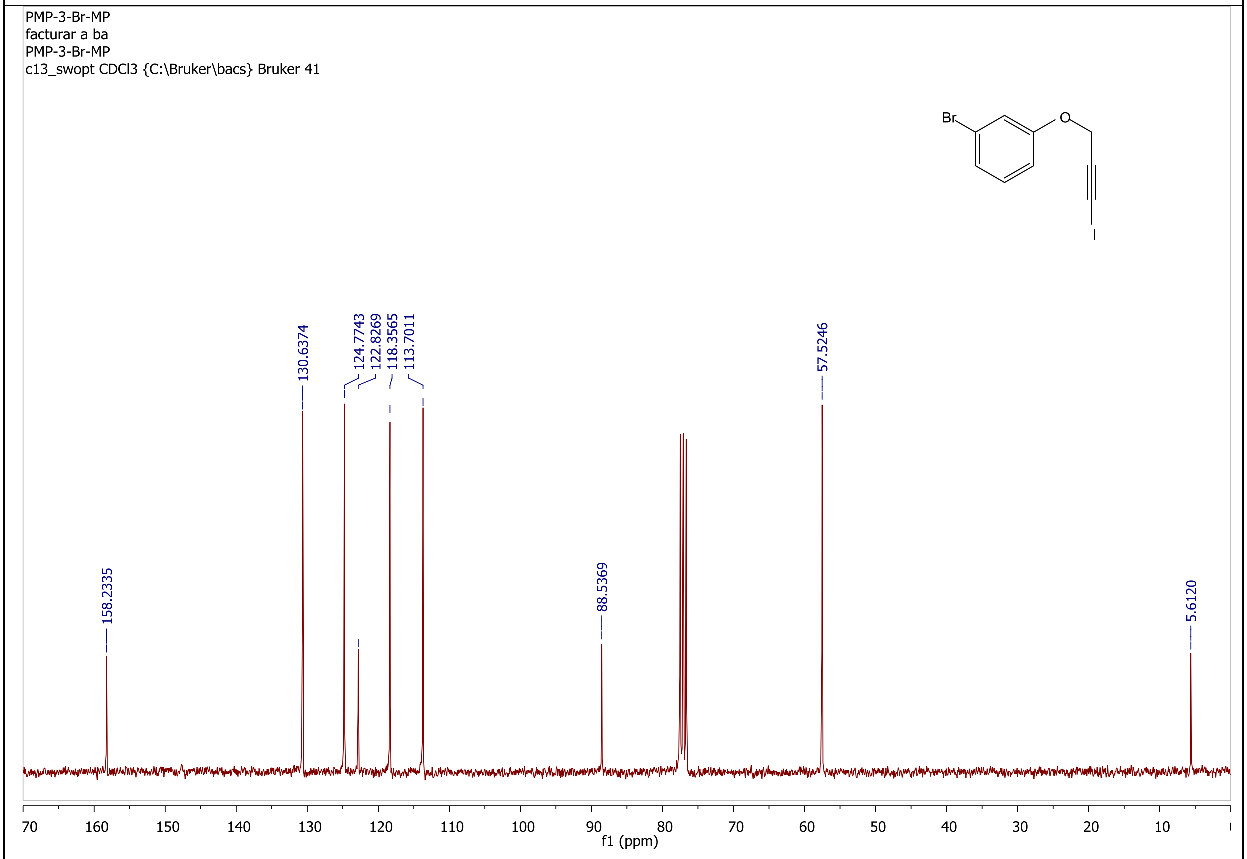
PMP-3-Br-MP
facturar a ba
PMP-3-Br-MP
c13_swopt CDCl3 {C:\Bruker\bacs} Bruker 41
Br
O
I
158.2335
130.6374
124.7743
122.8269
118.3565
113.7011
88.5369
57.5246
5.6120
f1 (ppm)

1h

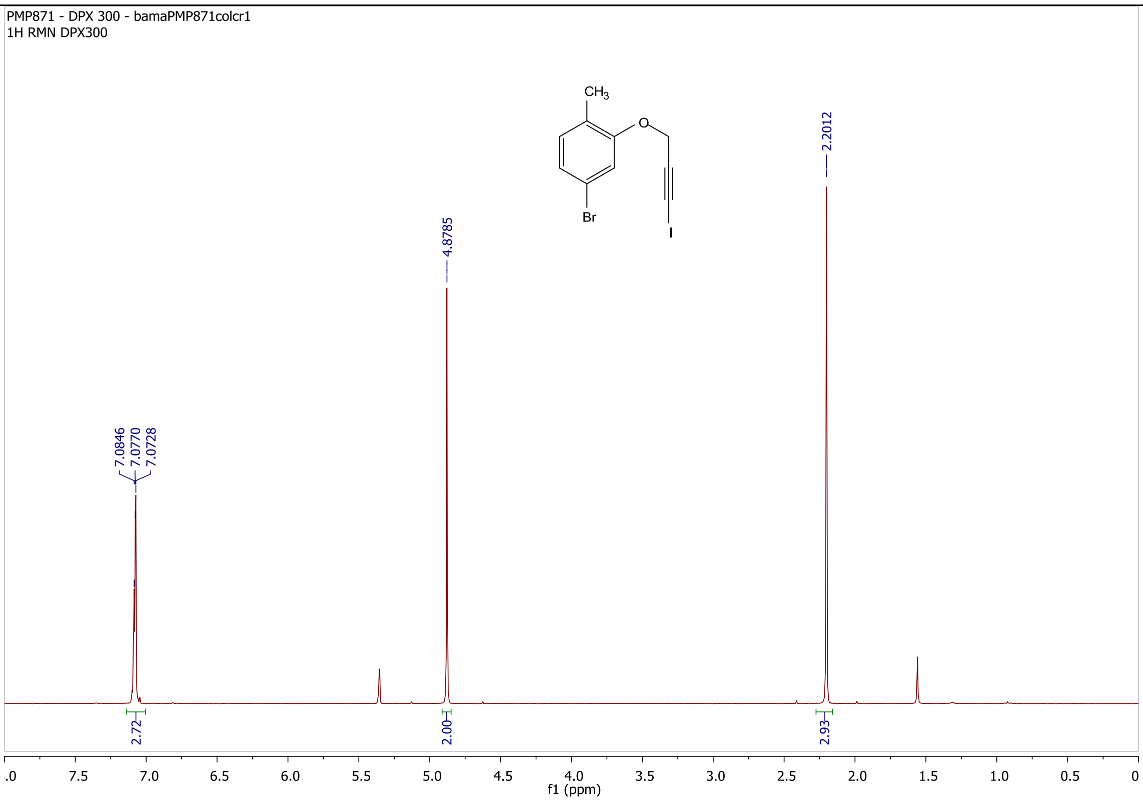
PMP871 - DPX 300 - bamaPMP871colcr1
1H RMN DPX300
CH3
O
Br
I
7.0846
7.0770
7.0728
4.8785
2.2012
2.72
2.00
2.93
f1 (ppm)

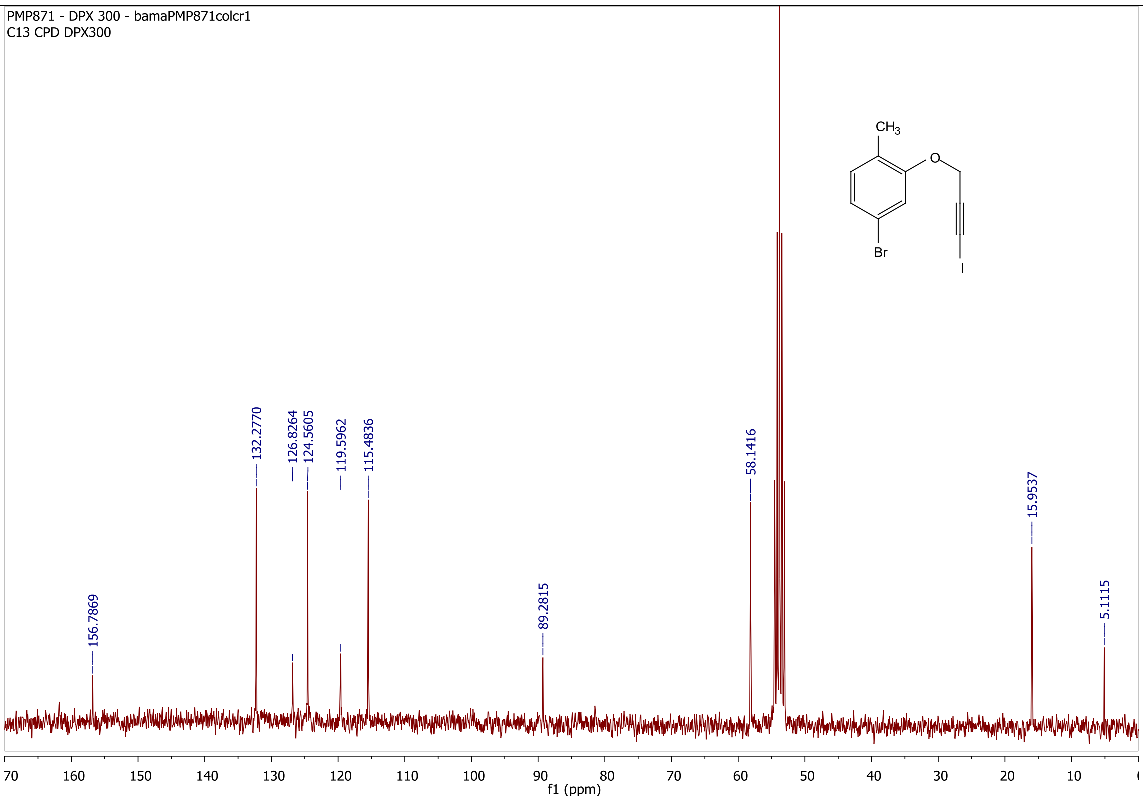
PMP871 - DPX 300 - bamaPMP871colcr1
C13 CPD DPX300
CH3
O
Br
I
156.7869
132.2770
126.8264
124.5605
119.5962
115.4836
89.2815
58.1416
15.9537
5.1115
f1 (ppm)

1i

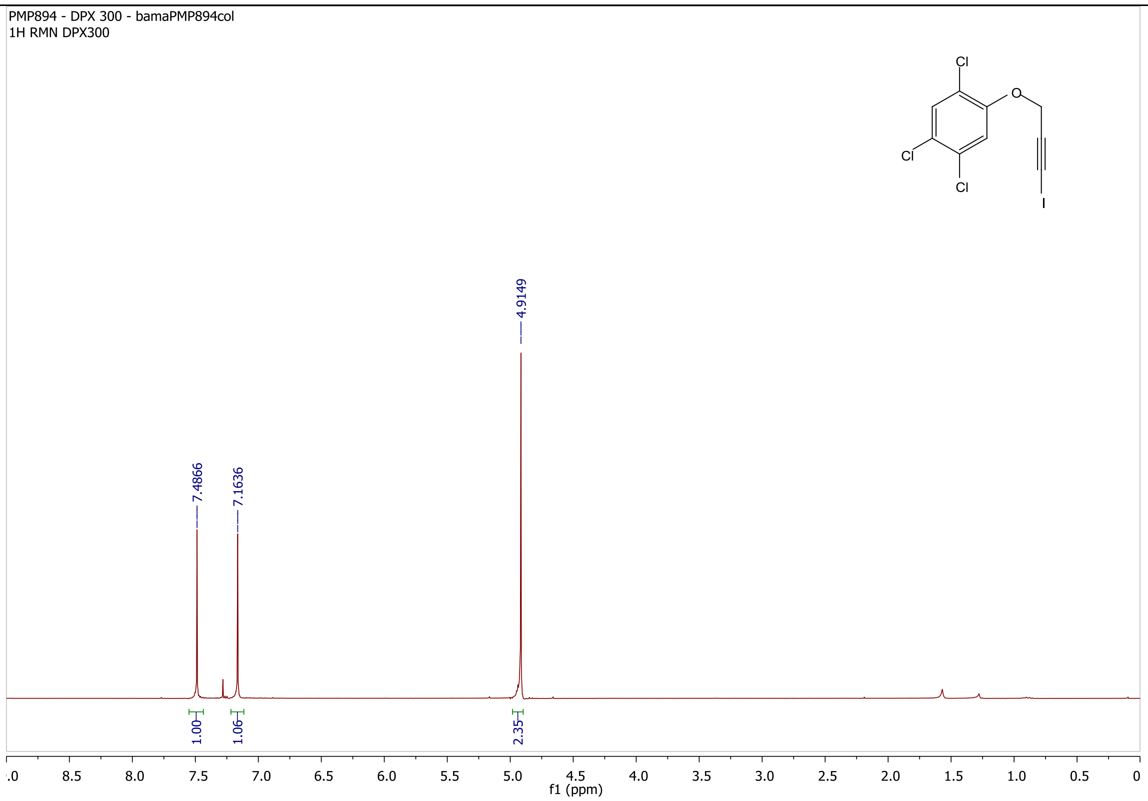

PMP894 - DPX 300 - bamaPMP894col
1H RMN DPX300
Cl
O
Cl
Cl
I
7.4866
7.1636
4.9149
1.00
1.06
2.35
f1 (ppm)

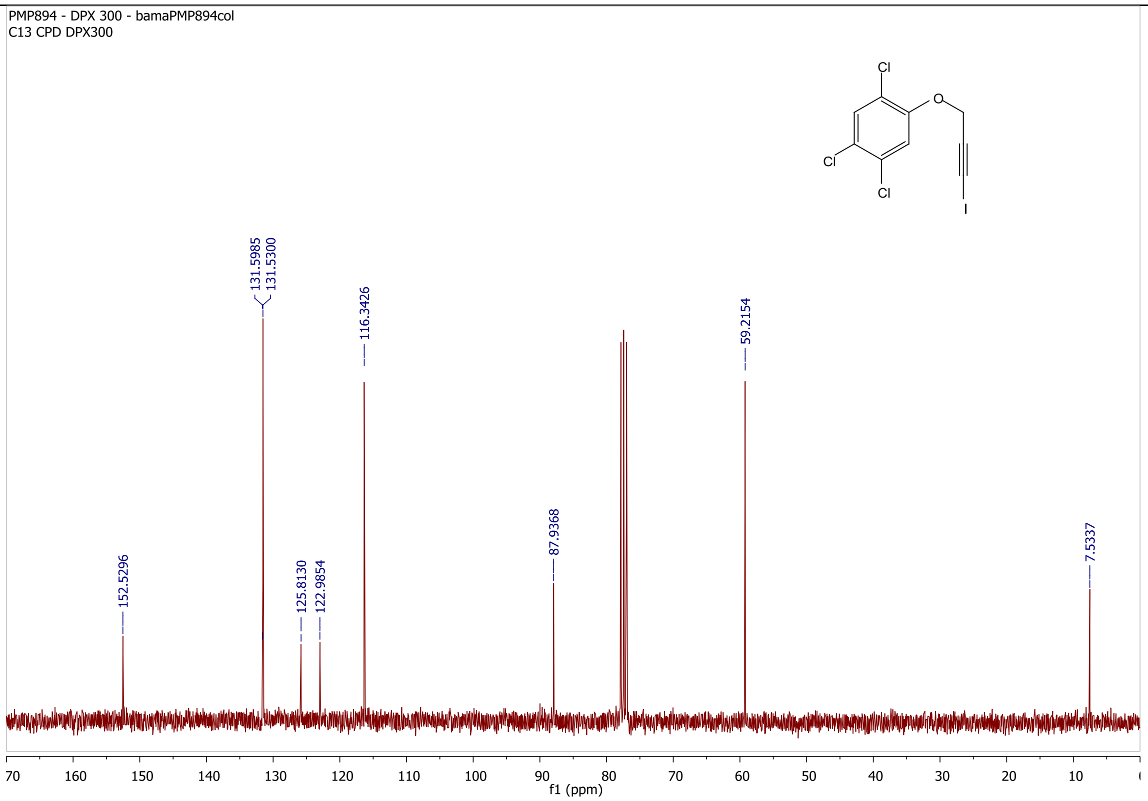

PMP894 - DPX 300 - bamaPMP894col
C13 CPD DPX300
Cl
O
Cl
Cl
I
131.5985
131.5300
116.3426
59.2154
152.5296
125.8130
122.9854
87.9368
7.5337
f1 (ppm)

1j

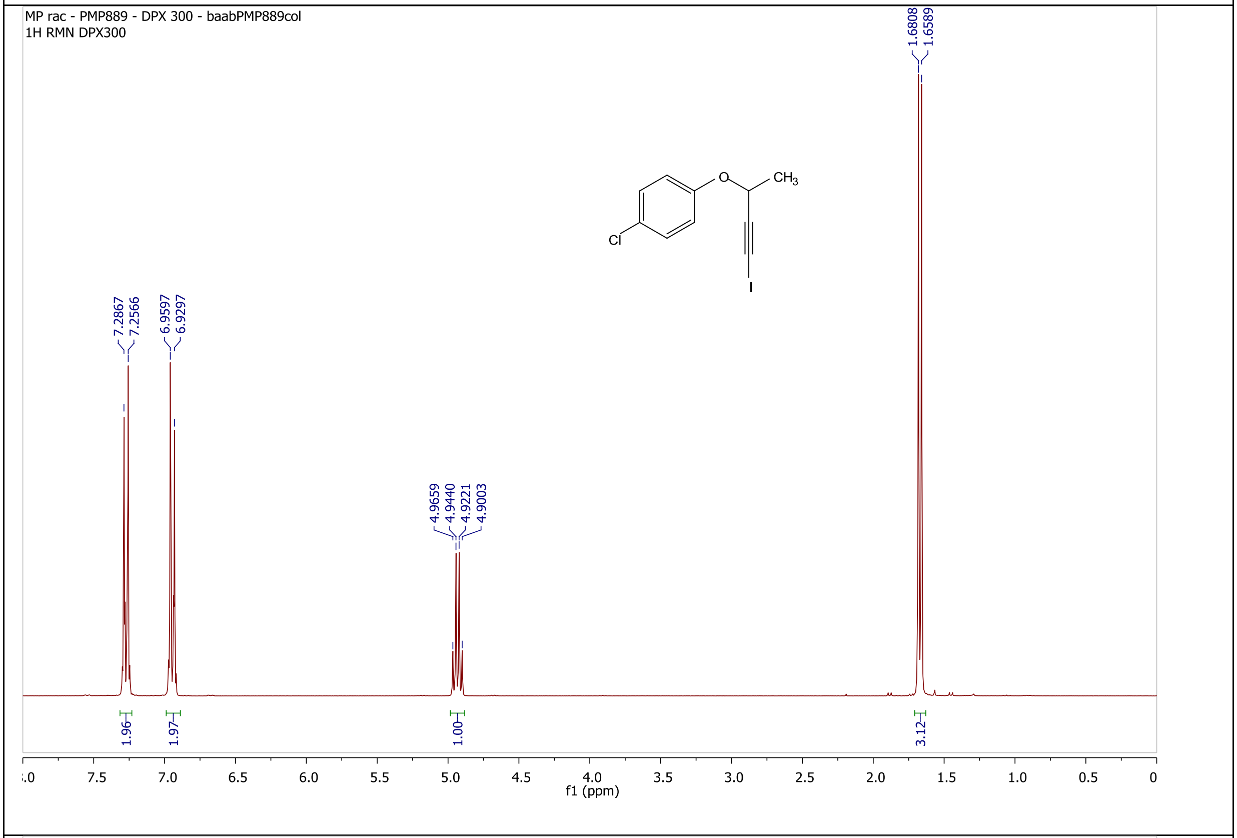

MP rac - PMP889 - DPX 300 - baabPMP889col
1H RMN DPX300
7.2867
7.2566
6.9597
6.9297
4.9659
4.9440
4.9221
4.9003
1.6808
1.6589
1.96
1.97
1.00
3.12
f1 (ppm)
CH3
O
Cl
I

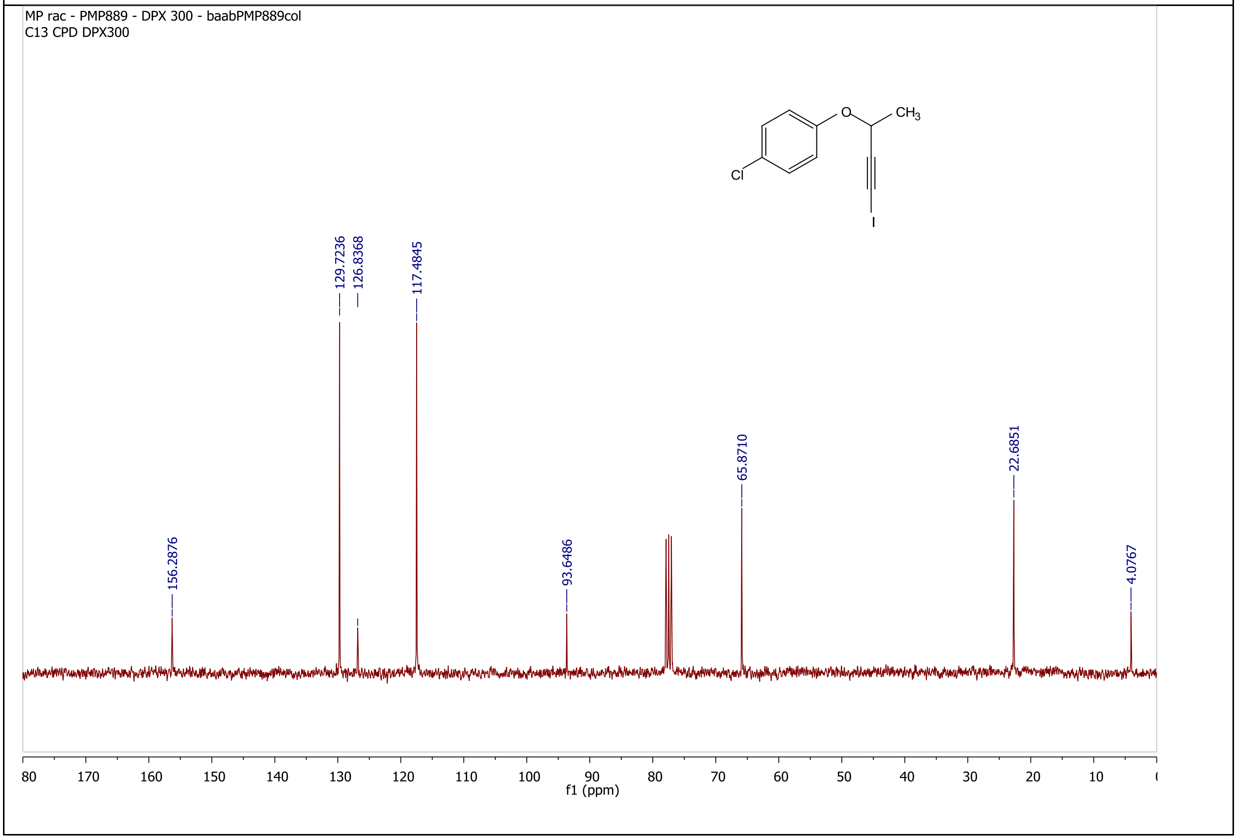

MP rac - PMP889 - DPX 300 - baabPMP889col
C13 CPD DPX300
156.2876
129.7236
126.8368
117.4845
93.6486
65.8710
22.6851
4.0767
f1 (ppm)
CH3
O
Cl
I

2a

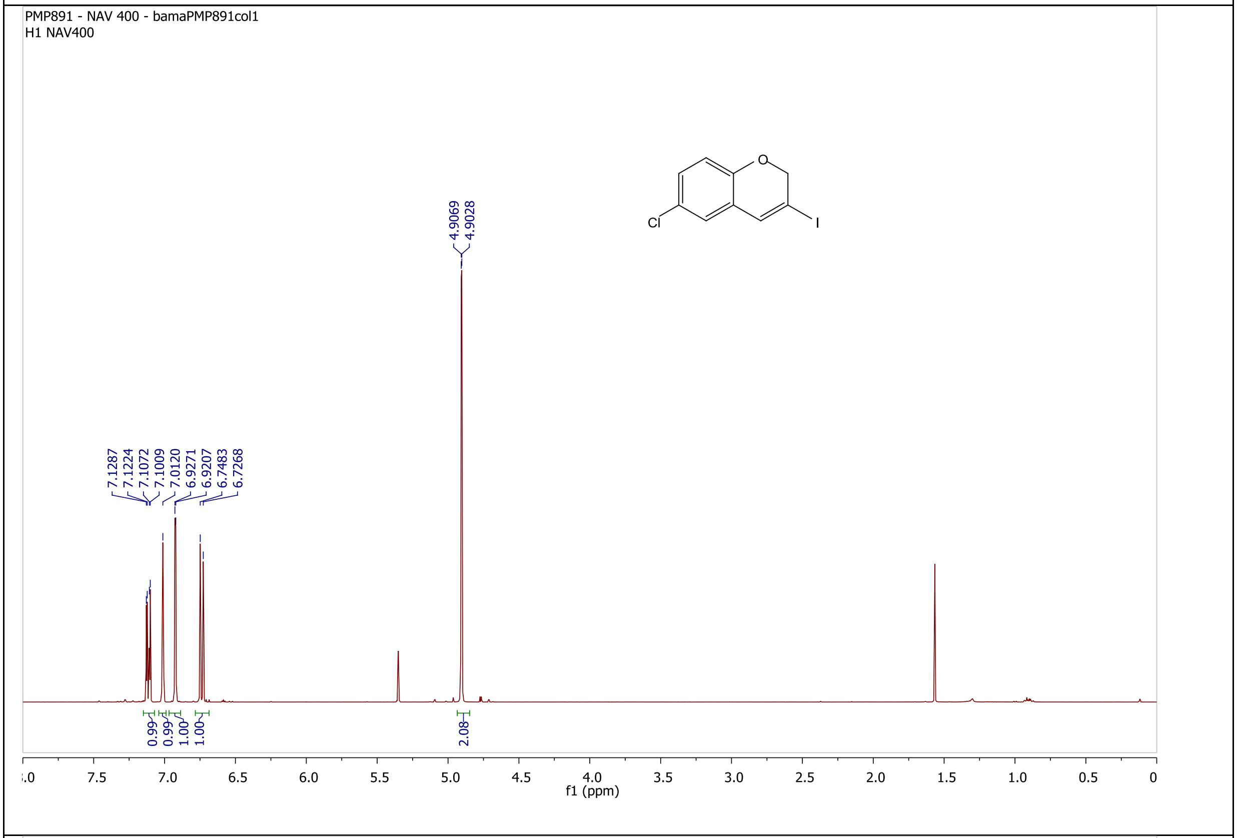
PMP891 - NAV 400 - bamaPMP891col1
H1 NAV400
7.1287
7.1224
7.1072
7.1009
7.0120
6.9271
6.9207
6.7483
6.7268
4.9069
4.9028
0.99
0.99
1.00
1.00
2.08
f1 (ppm)
Cl
O
I

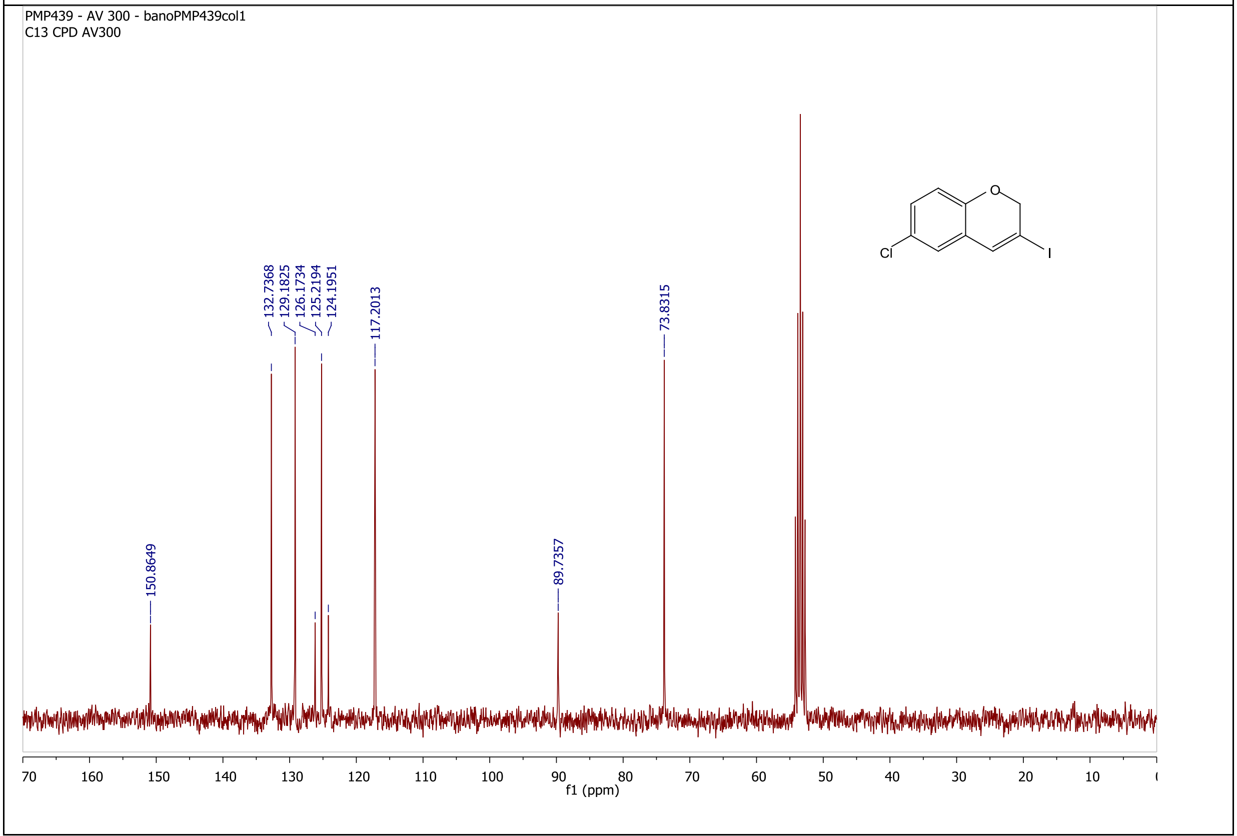
PMP439 - AV 300 - banoPMP439col1
C13 CPD AV300
150.8649
132.7368
129.1825
126.1734
125.2194
124.1951
117.2013
89.7357
73.8315
f1 (ppm)
Cl
O
I

2b

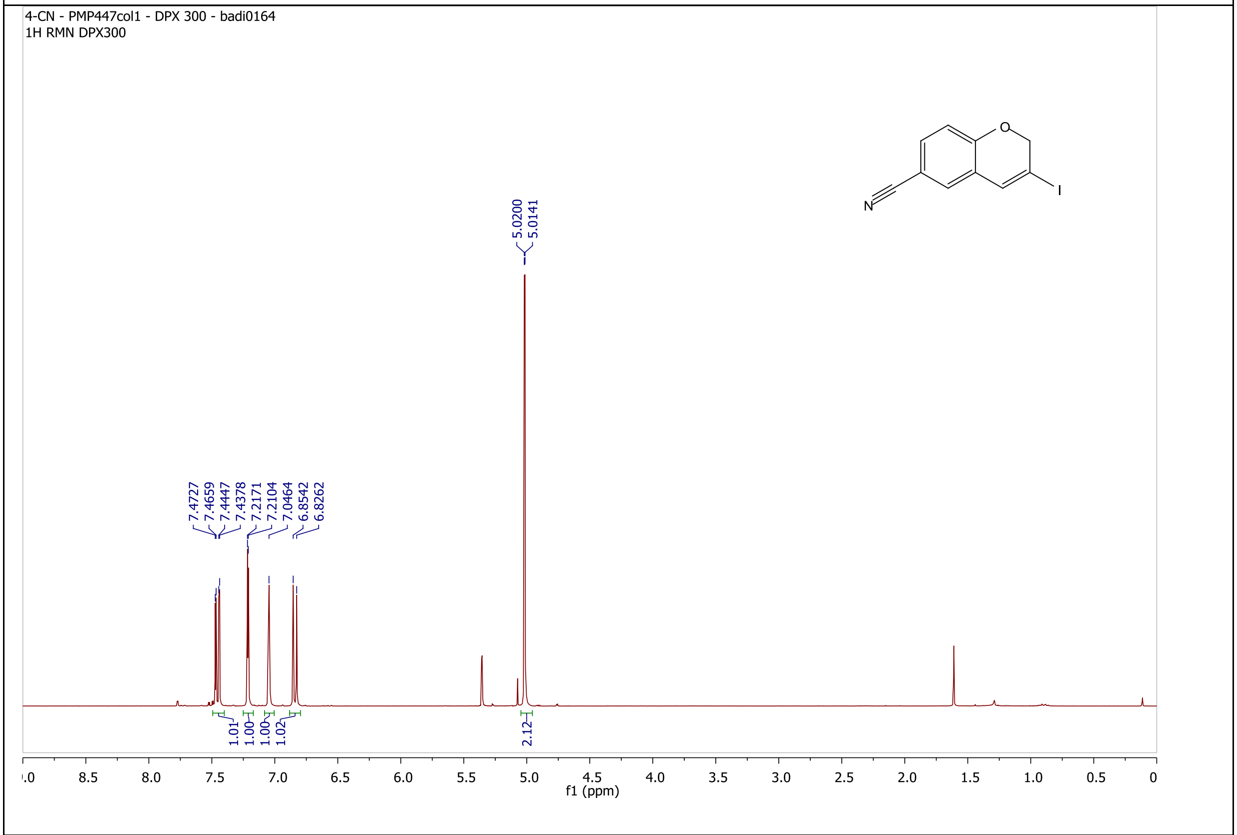
4-CN - PMP447col1 - DPX 300 - badi0164
1H RMN DPX300
7.4727
7.4659
7.4447
7.4378
7.2171
7.2104
7.0464
6.8542
6.8262
5.0200
5.0141
1.01
1.00
1.00
1.02
2.12
f1 (ppm)
O
N
I

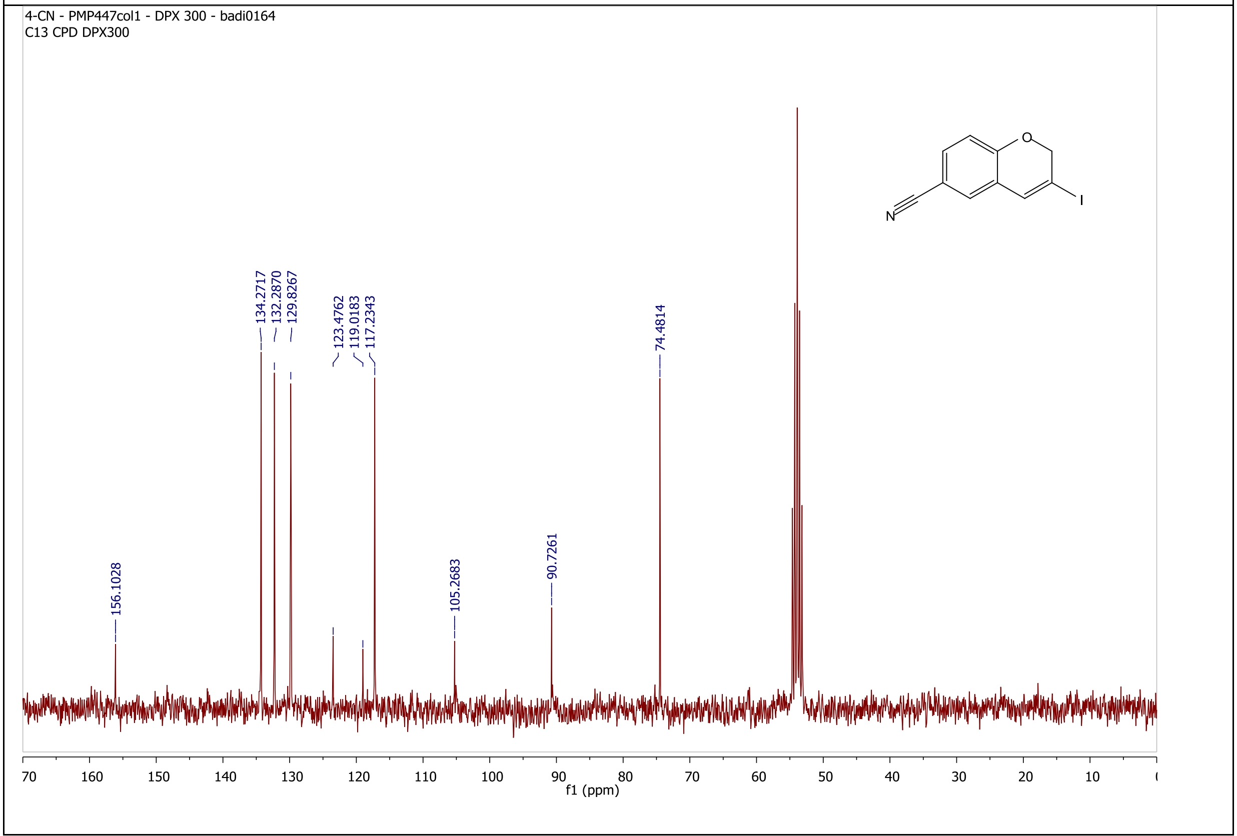
4-CN - PMP447col1 - DPX 300 - badi0164
C13 CPD DPX300
156.1028
134.2717
132.2870
129.8267
123.4762
119.0183
117.2343
105.2683
90.7261
74.4814
f1 (ppm)
O
N
I

2c

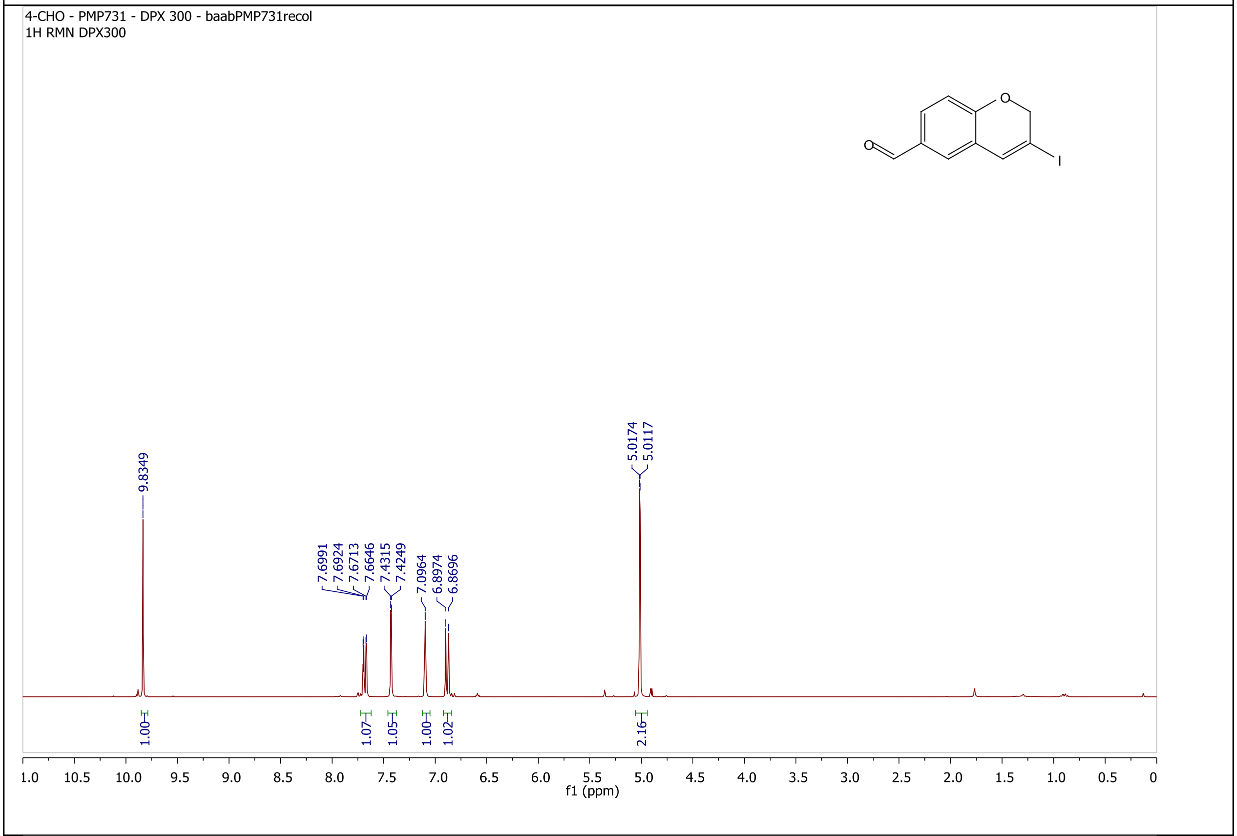

4-CHO - PMP731 - DPX 300 - baabPMP731recol
1H RMN DPX300
9.8349
7.6991
7.6924
7.6713
7.6646
7.4315
7.4249
7.0964
6.8974
6.8696
5.0174
5.0117
1.00
1.07
1.05
1.00
1.02
2.16
f1 (ppm)

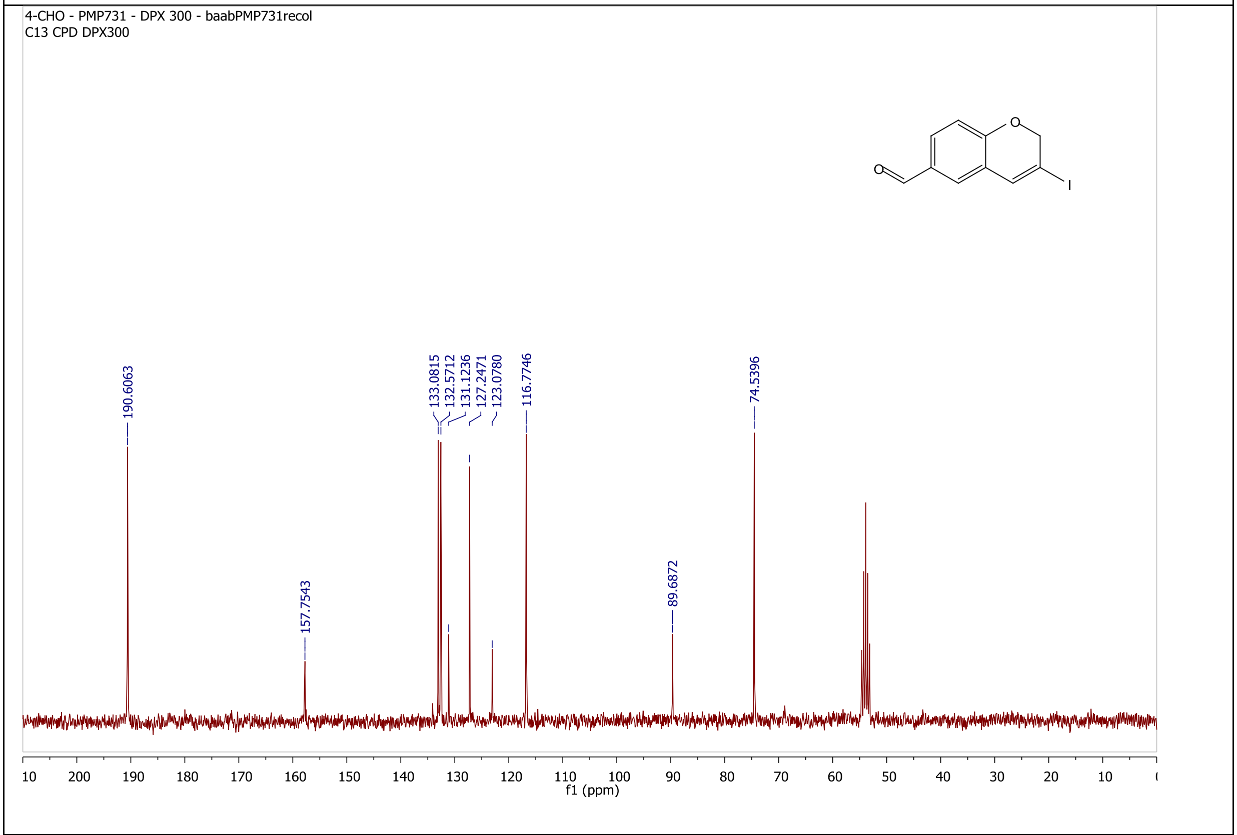

4-CHO - PMP731 - DPX 300 - baabPMP731recol
C13 CPD DPX300
190.6063
157.7543
133.0815
132.5712
131.1236
127.2471
123.0780
116.7746
89.6872
74.5396
f1 (ppm)

2d

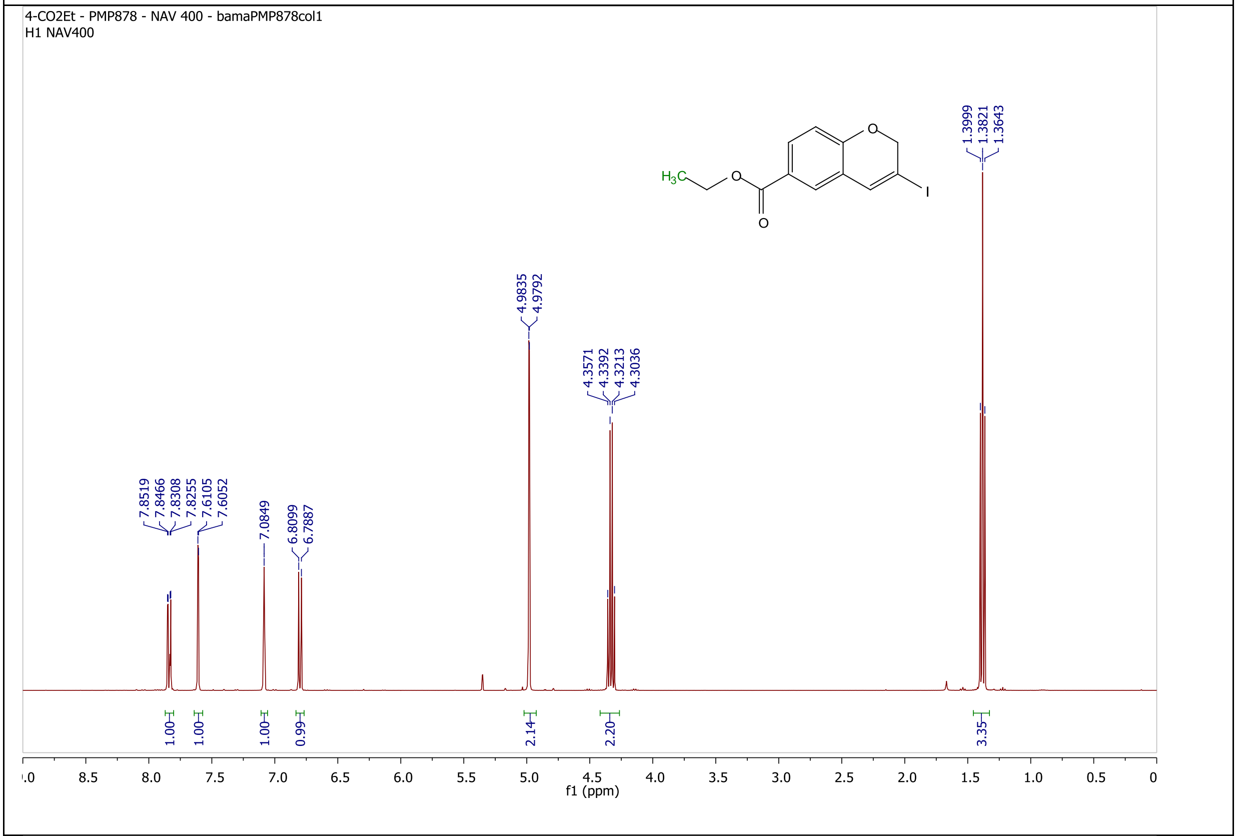

4-CO2Et - PMP878 - NAV 400 - bamaPMP878col1
H1 NAV400
H3C
O
O
O
I
7.8519
7.8466
7.8308
7.8255
7.6105
7.6052
7.0849
6.8099
6.7887
4.9835
4.9792
4.3571
4.3392
4.3213
4.3036
1.3999
1.3821
1.3643
1.00
1.00
1.00
0.99
2.14
2.20
3.35
f1 (ppm)

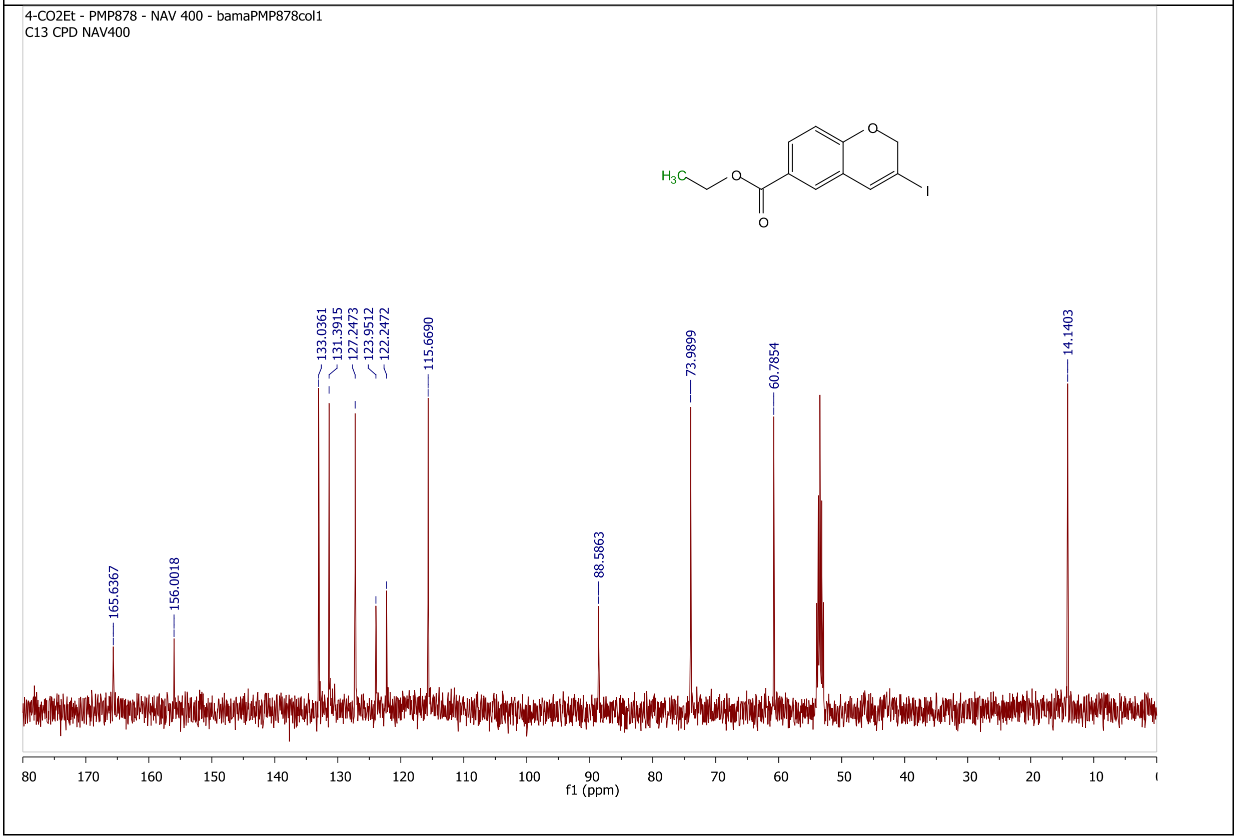

4-CO2Et - PMP878 - NAV 400 - bamaPMP878col1
C13 CPD NAV400
H3C
O
O
O
I
165.6367
156.0018
133.0361
131.3915
127.2473
123.9512
122.2472
115.6690
88.5863
73.9899
60.7854
14.1403
f1 (ppm)

2e

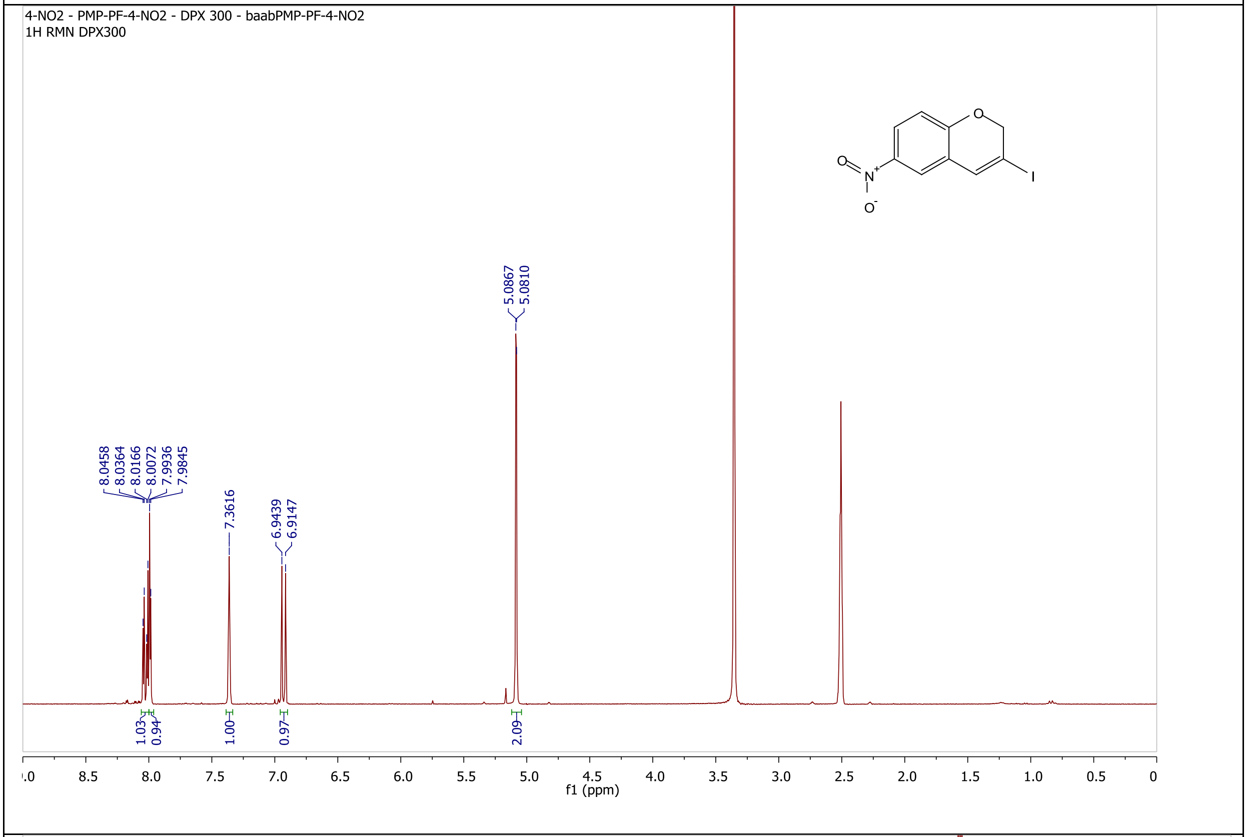
4-NO2 - PMP-PF-4-NO2 - DPX 300 - baabPMP-PF-4-NO2
1H RMN DPX300
8.0458
8.0364
8.0166
8.0072
7.9936
7.9845
7.3616
6.9439
6.9147
5.0867
5.0810
1.03
0.94
1.00
0.97
2.09
f1 (ppm)
O
N+
O-
I

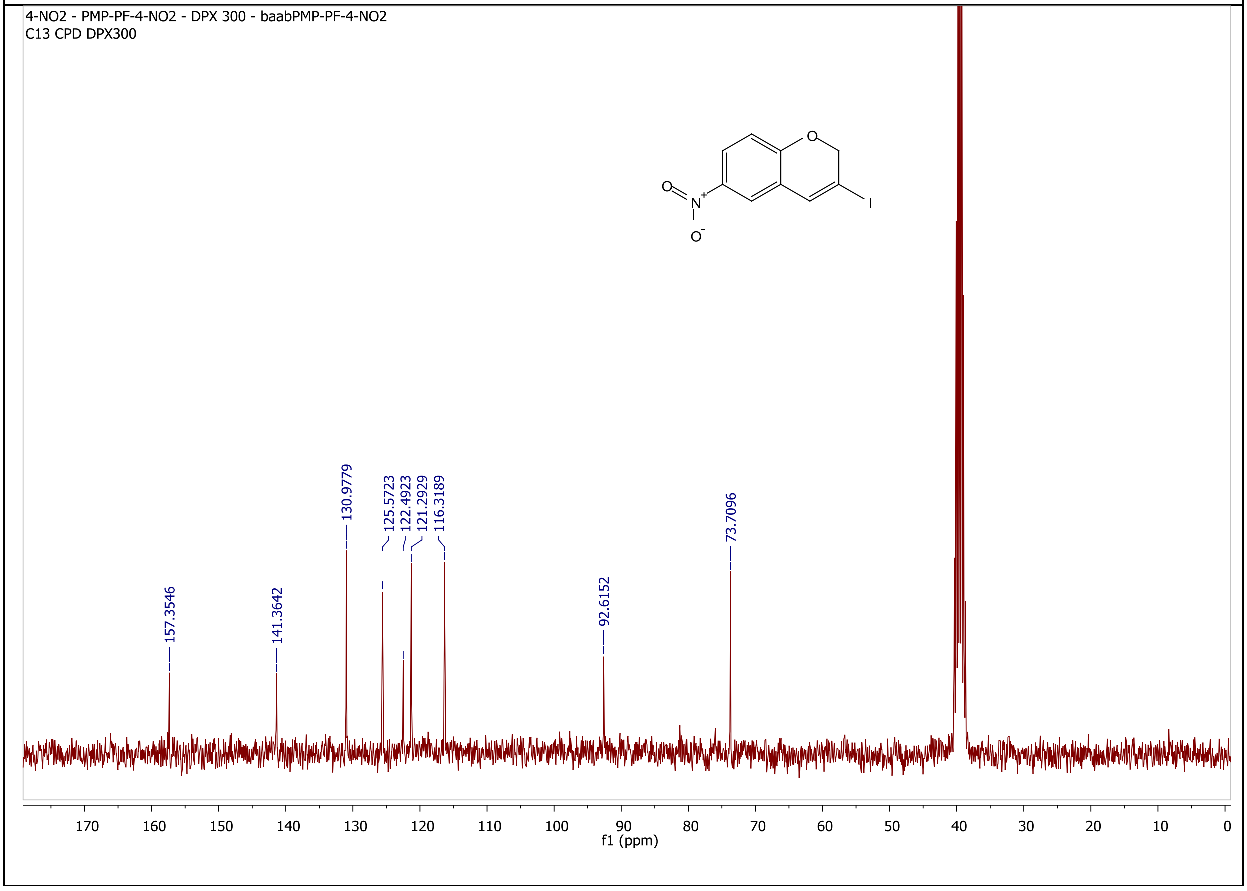
4-NO2 - PMP-PF-4-NO2 - DPX 300 - baabPMP-PF-4-NO2
C13 CPD DPX300
157.3546
141.3642
130.9779
125.5723
122.4923
121.2929
116.3189
92.6152
73.7096
f1 (ppm)
O
N+
O-
I

2f

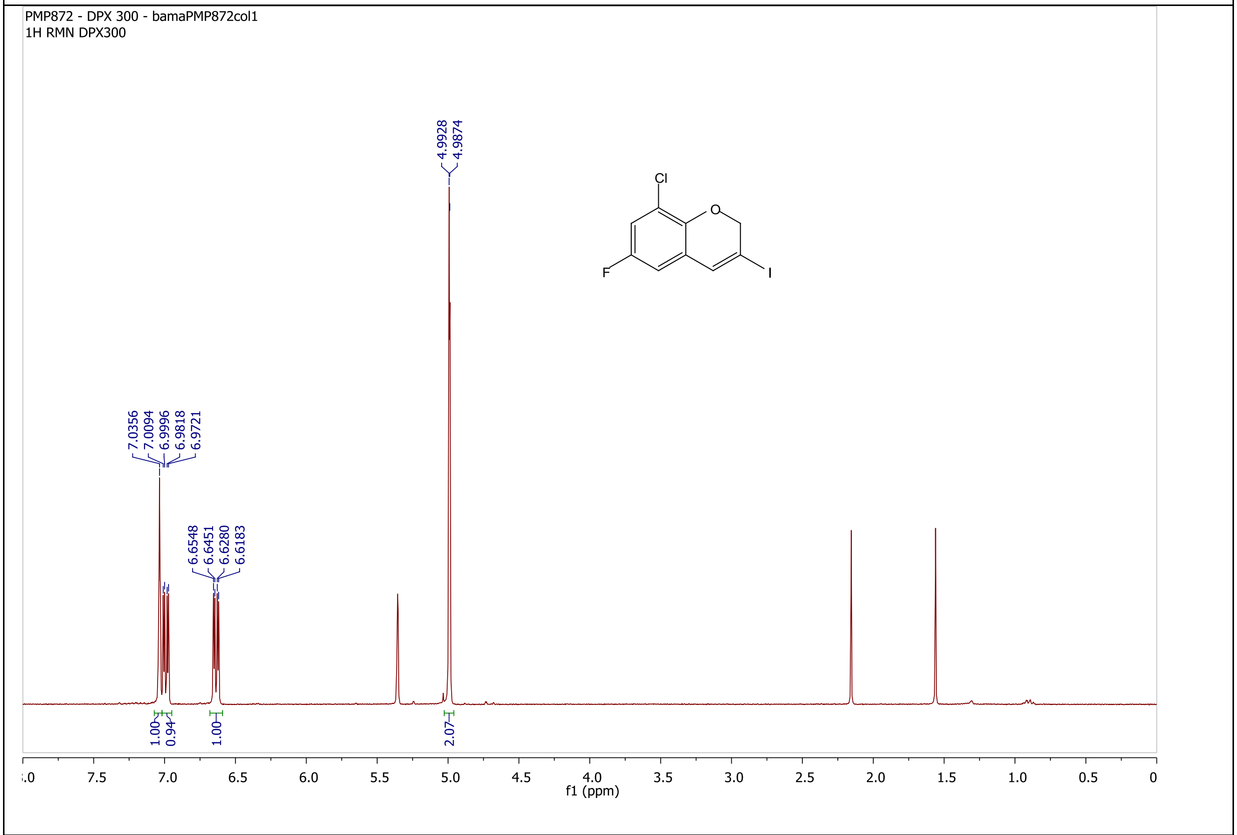
PMP872 - DPX 300 - bamaPMP872col1
1H RMN DPX300
4.9928
4.9874
7.0356
7.0094
6.9996
6.9818
6.9721
6.6548
6.6451
6.6280
6.6183
1.00
0.94
1.00
2.07
f1 (ppm)
Cl
O
F
I

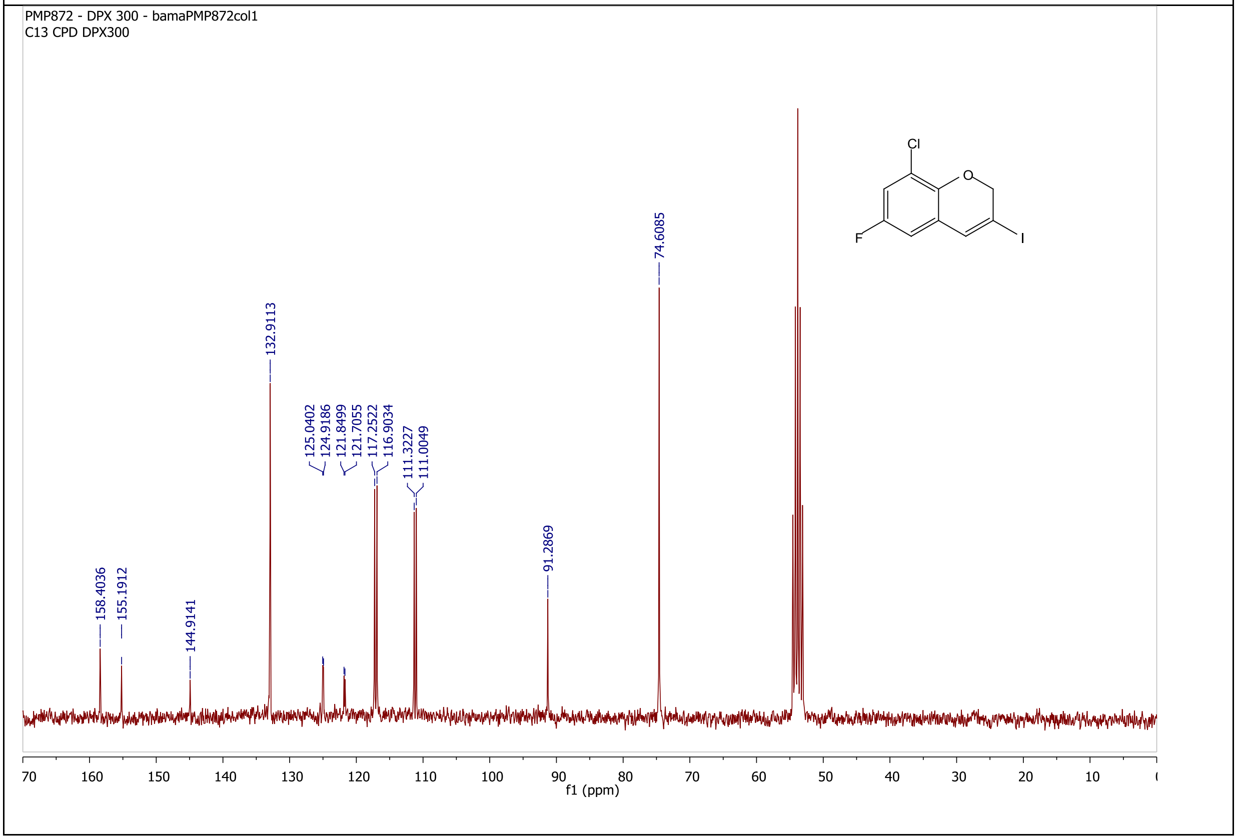
PMP872 - DPX 300 - bamaPMP872col1
C13 CPD DPX300
158.4036
155.1912
144.9141
132.9113
125.0402
124.9186
121.8499
121.7055
117.2522
116.9034
111.3227
111.0049
91.2869
74.6085
f1 (ppm)
Cl
O
F
I

2g + 2g’

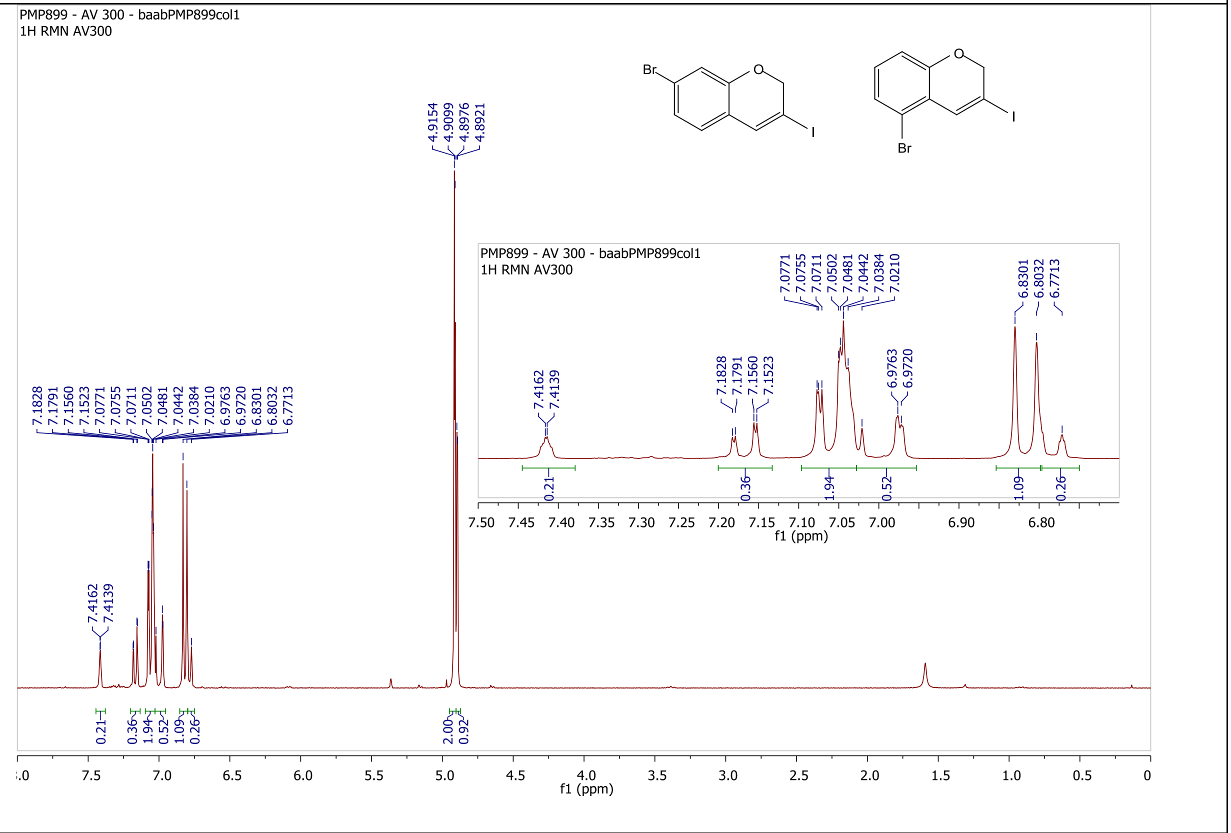
PMP899 - AV 300 - baabPMP899col1
1H RMN AV300
Br
O
I
Br
O
I
4.9154
4.9099
4.8976
4.8921
7.1828
7.1791
7.1560
7.1523
7.0771
7.0755
7.0711
7.0502
7.0481
7.0442
7.0384
7.0210
6.9763
6.9720
6.8301
6.8032
6.7713
7.4162
7.4139
0.21
0.36
1.94
0.52
1.09
0.26
2.00
0.92
f1 (ppm)

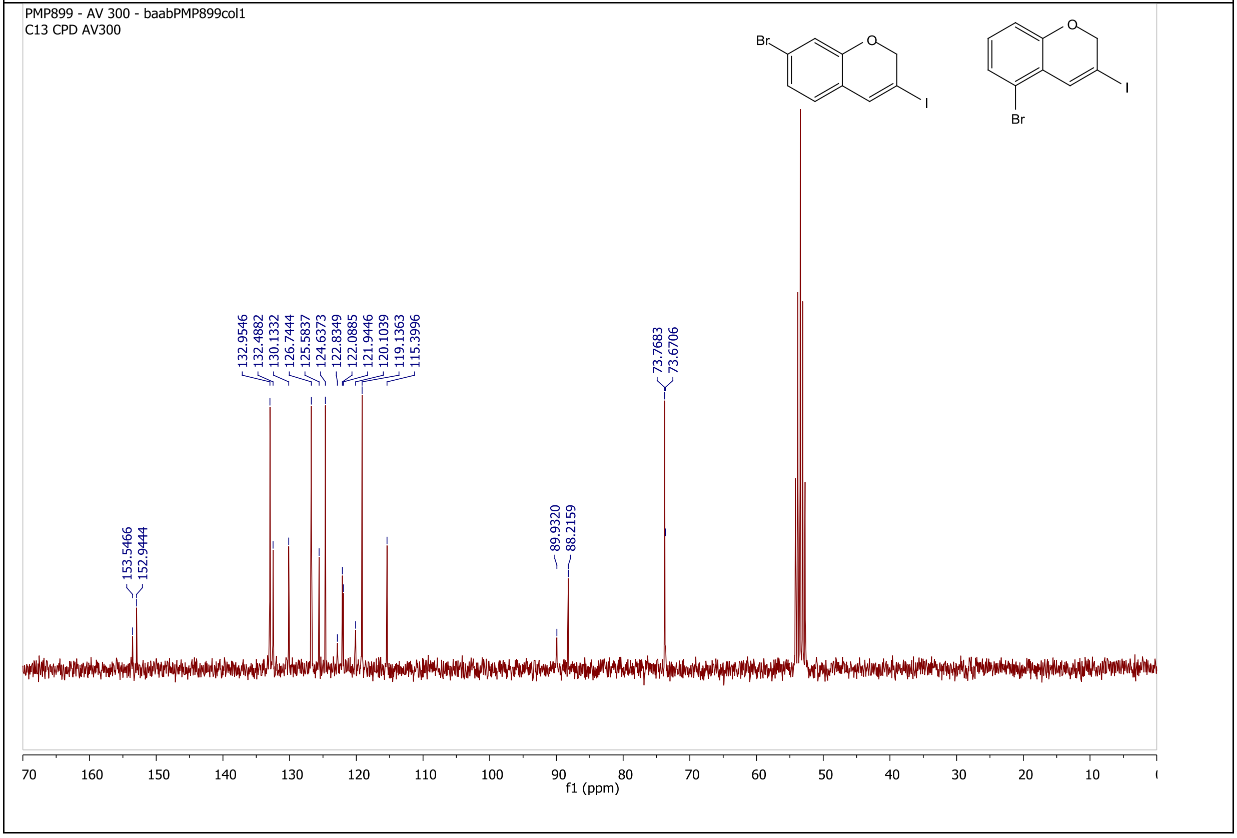
PMP899 - AV 300 - baabPMP899col1
C13 CPD AV300
Br
O
I
Br
O
I
132.9546
132.4882
130.1332
126.7444
125.5837
124.6373
122.8349
122.0885
121.9446
120.1039
119.1363
115.3996
73.7683
73.6706
89.9320
88.2159
153.5466
152.9444
f1 (ppm)

2h

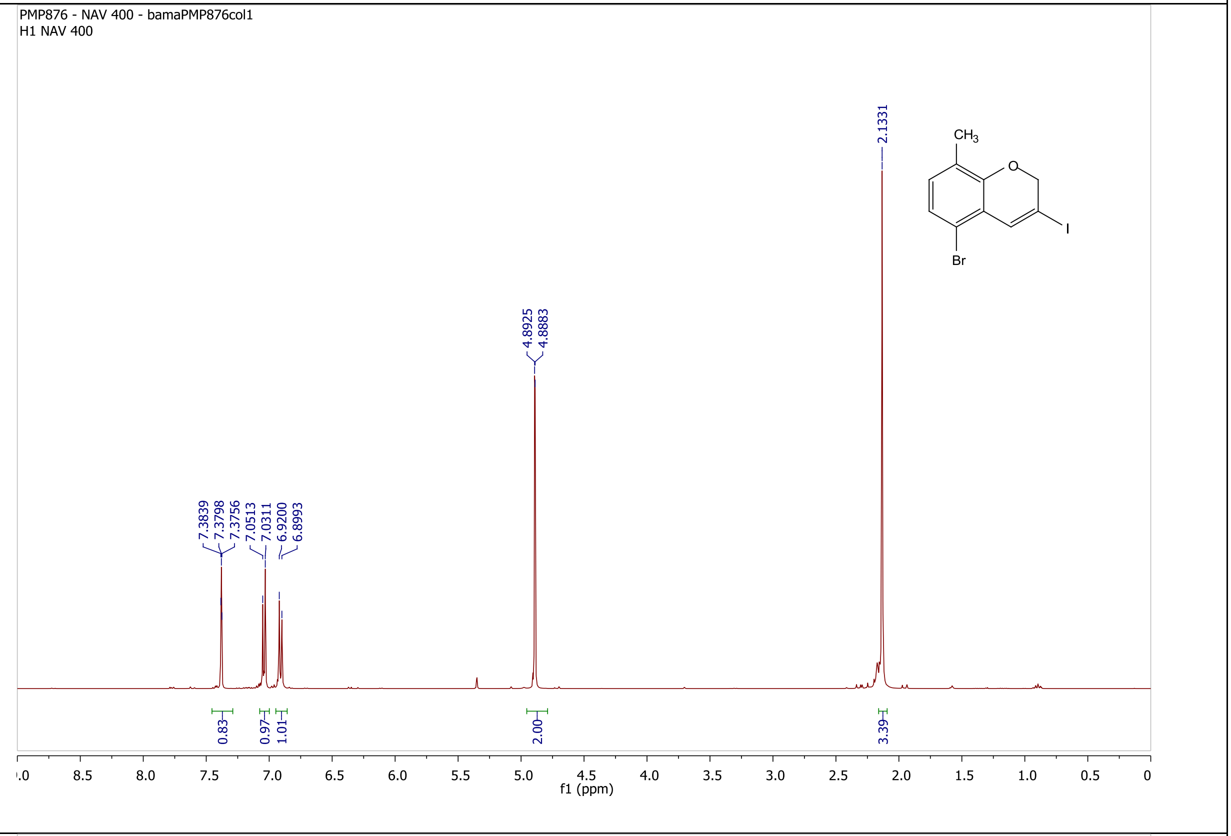
PMP876 - NAV 400 - bamaPMP876col1
H1 NAV 400
7.3839
7.3798
7.3756
7.0513
7.0311
6.9200
6.8993
4.8925
4.8883
2.1331
0.83
0.97
1.01
2.00
3.39
CH3
O
I
Br
f1 (ppm)

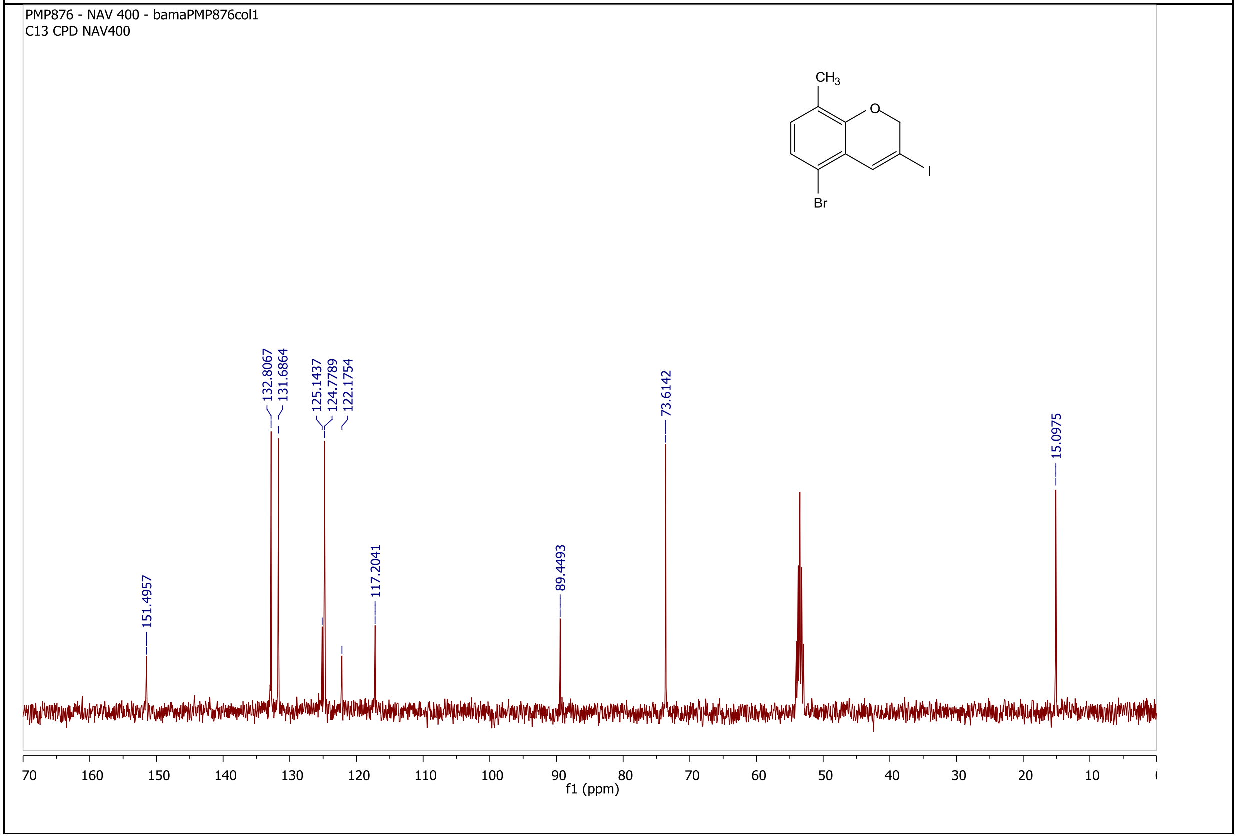
PMP876 - NAV 400 - bamaPMP876col1
C13 CPD NAV400
151.4957
132.8067
131.6864
125.1437
124.7789
122.1754
117.2041
89.4493
73.6142
15.0975
CH3
O
I
Br
f1 (ppm)

2i

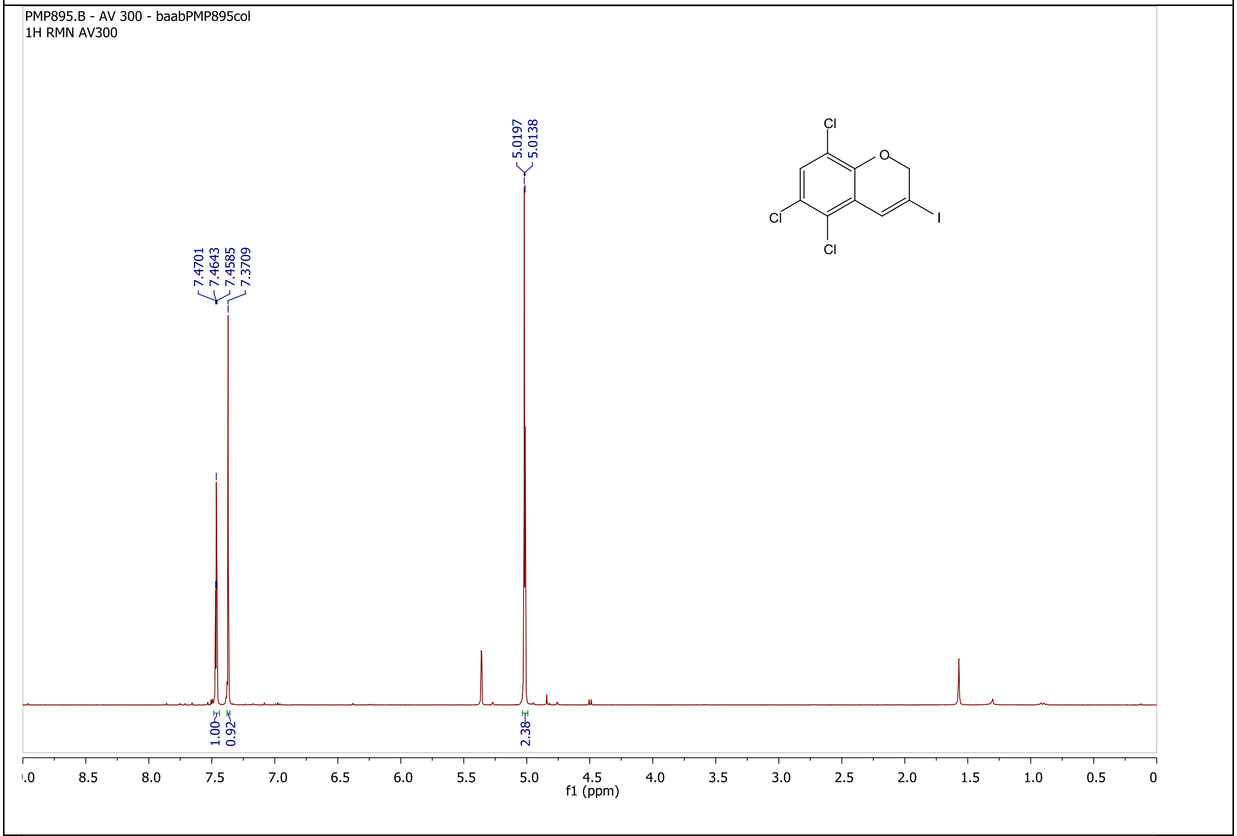
PMP895.B - AV 300 - baabPMP895col
1H RMN AV300
7.4701
7.4643
7.4585
7.3709
5.0197
5.0138
1.00
0.92
2.38
Cl
O
Cl
I
Cl
f1 (ppm)

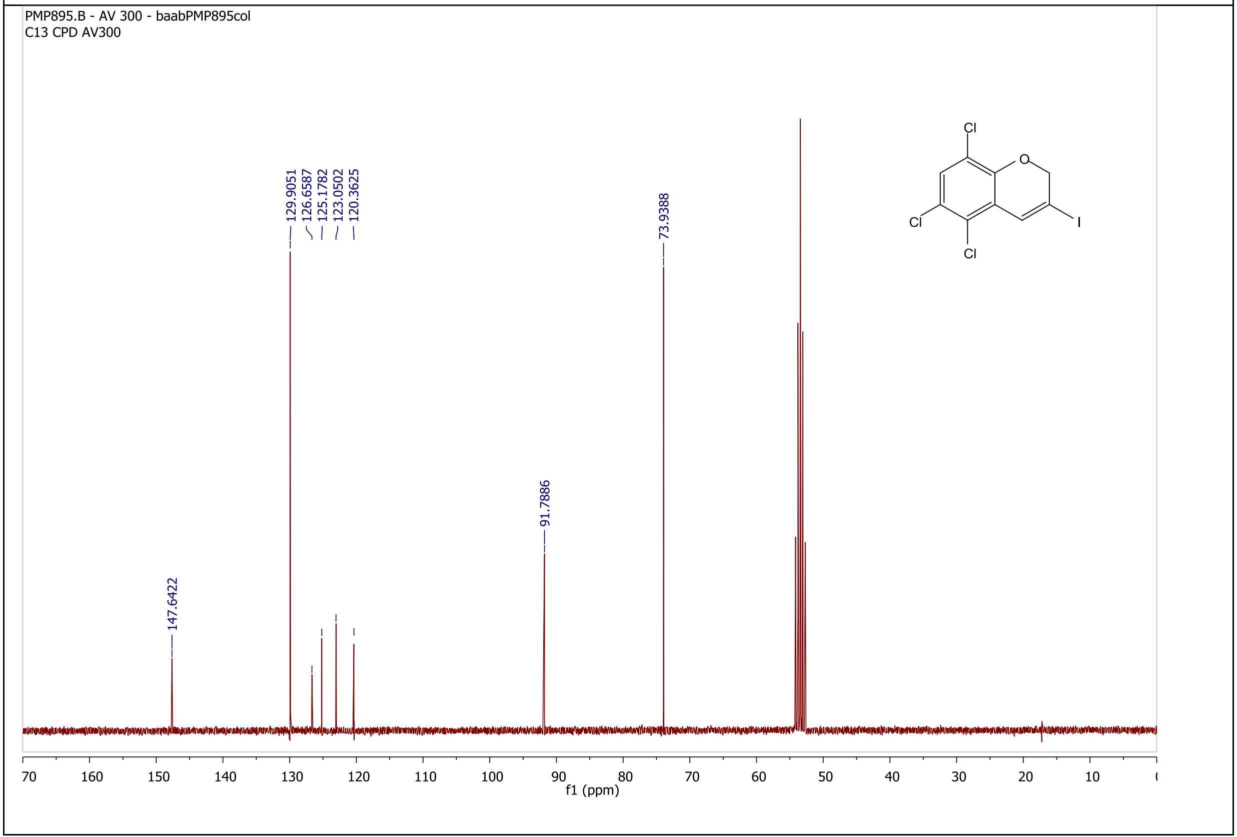
PMP895.B - AV 300 - baabPMP895col
C13 CPD AV300
129.9051
126.6587
125.1782
123.0502
120.3625
73.9388
91.7886
147.6422
Cl
O
Cl
I
Cl
f1 (ppm)

2j

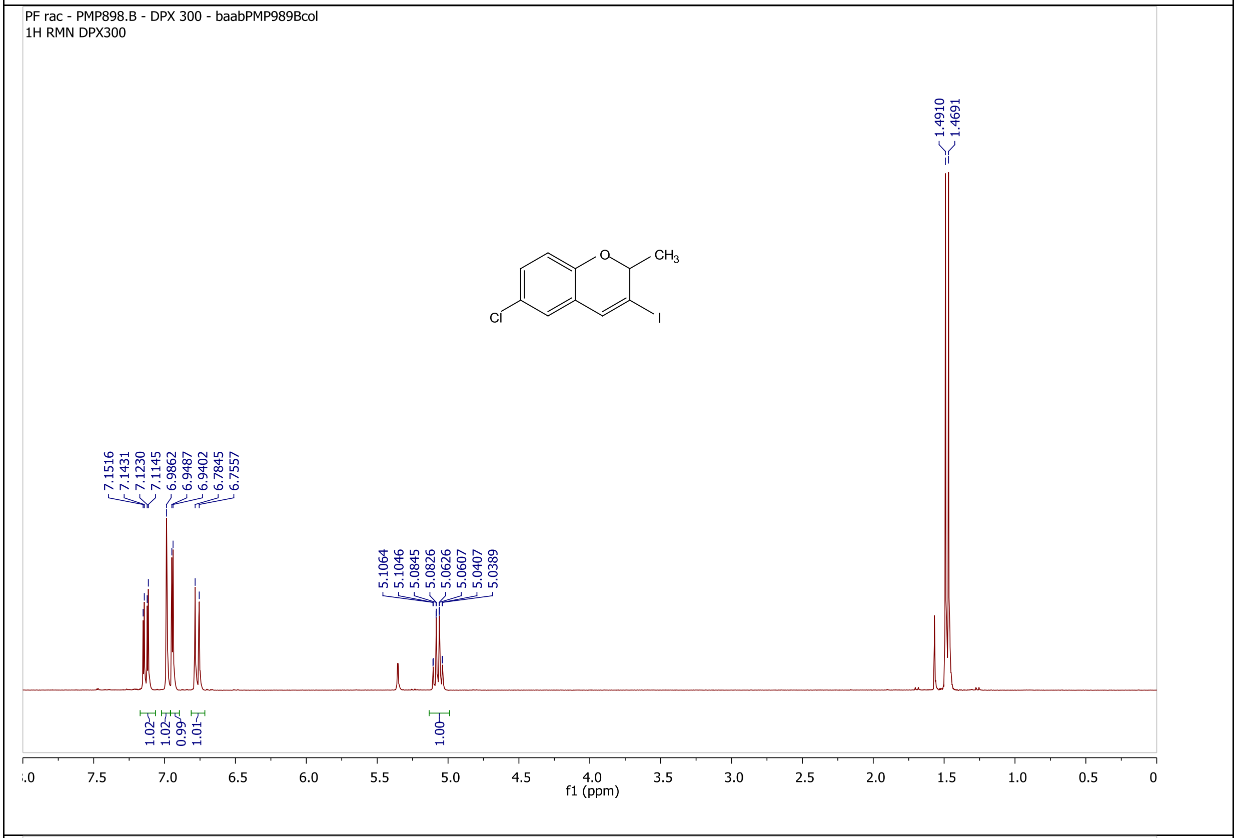
PF rac - PMP898.B - DPX 300 - baabPMP989Bcol
1H RMN DPX300
7.1516
7.1431
7.1230
7.1145
6.9862
6.9487
6.9402
6.7845
6.7557
5.1064
5.1046
5.0845
5.0826
5.0626
5.0607
5.0407
5.0389
1.4910
1.4691
1.02
1.02
0.99
1.01
1.00
f1 (ppm)
O
CH3
Cl
I

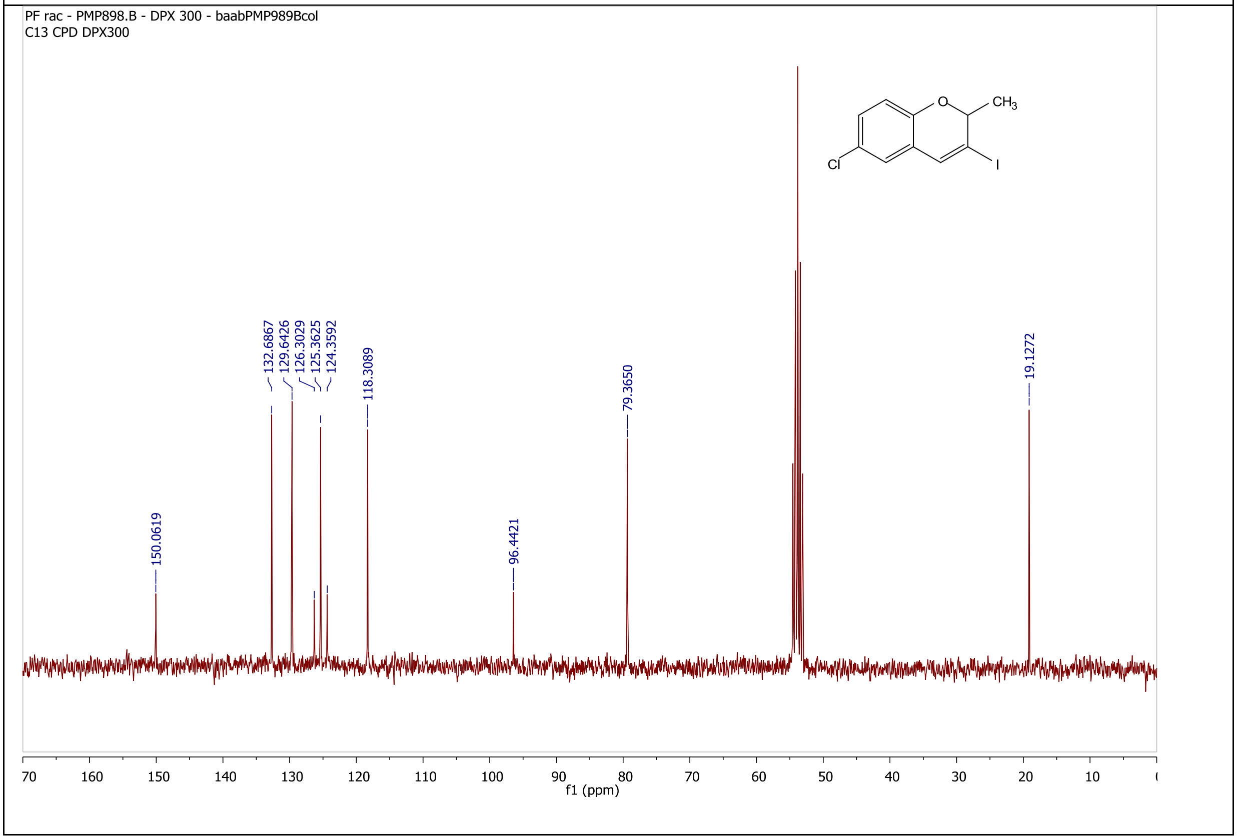
PF rac - PMP898.B - DPX 300 - baabPMP989Bcol
C13 CPD DPX300
150.0619
132.6867
129.6426
126.3029
125.3625
124.3592
118.3089
96.4421
79.3650
19.1272
f1 (ppm)
O
CH3
Cl
I

3. X-ray molecular structure for **2f**

CCDC 939930 contains the supplementary crystallographic data for this paper. These data can be obtained free of charge from the Cambridge Crystallographic Data Centre via www.cam.ac.uk/data_request/cif.

The most relevant crystal and refinement data for *8-chloro-6-fluoro-3-iodo-2H-chromene* is as follows:

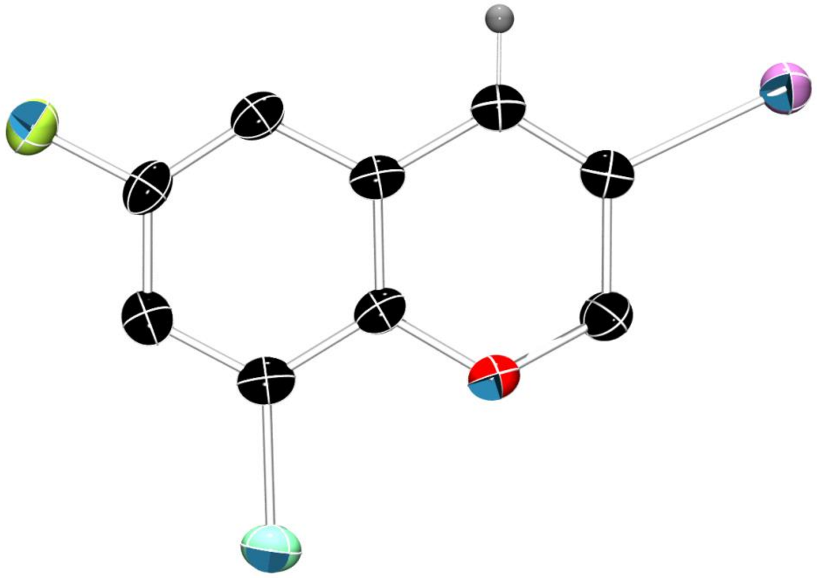

Empirical formula C_9H_5ClFIO, M_r = 310.49, T = 173 (2) K, λ =0.71073 Å, crystal system, space group: triclinic, 'P -1', unit cell dimensions: *a* = 4.3434 (3), *b* = 9.8103 (9), *c* = 11.5211 (10) Å, α = 111.092 (3), β = 91.559 (5), γ = 93.498 (5) °, V = 456.53 (7) Å, Z = 2, ρ_{calcd} = 2.259 gcm^{-1}, μ = 3.767 mm^{-1}, *F*(000) = 292 crystal si : 0.45 x 0.30 x 0.10 mm, θrange data collection : 4.17 – 27.48 °, index ranges: $-5 \leq h \leq 5$, $-12 \leq k \leq 11$, $-0 \leq l \leq 14$, reflections collected/unique = 2071/2071 [R_{int} = 0.0000], completeness to 2θ = 27.48 (98.4 %), absorption correction: semi-empirical from equivalents, max. and min. transmission = 0.7103 and 0.1494 , refinement method: full matrix least-squares on F^2 , data/restraints/parameters = 2071/0/118, goodness-of-fit on F^2 = 1.058, final *R* indices [$I>2\sigma(I)$]: R_1 = 0.0336, wR_2 = 0.0855, R indices (all data): R_1 = 0.0389, wR_2 = 0.0879; largest difference peak and hole = 0.896 and -1.021 e Å^{-3}.

4. HPLC chromatograms for **1j** and **2j**

The compound (*R*)-**1j** was obtained from commercially available (*S*)-3-butyn-2-ol using standard Mitsunobu-type chemistry, which is known to yield the desired aryl-substituted ether arising from clean inversion of configuration.

HPLC Chromatogram for (*R*)-1-chloro-4-((4-iodobut-3-yn-2-yl)oxy)benzene

CHIRALCEL OD-H: *n*-Hexane: Isopropanol; 99:1; Flow 0.4 ml/min; λ = 226.0 nm

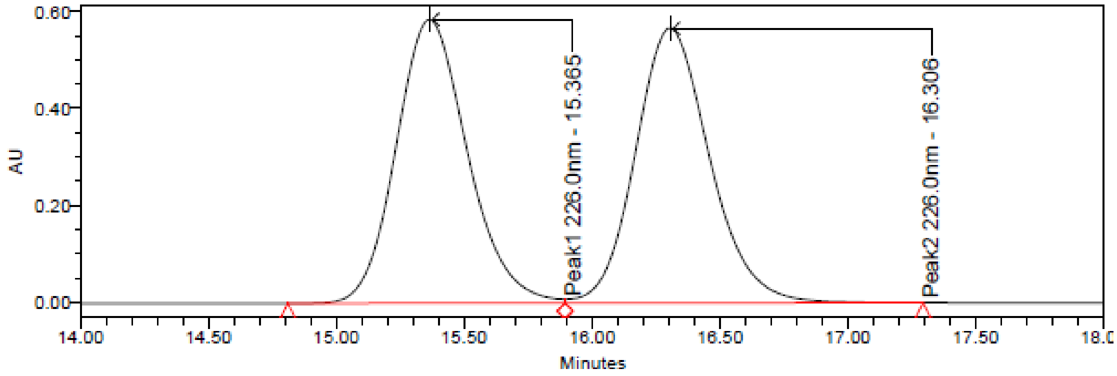

Peak Results

	Name	RT	Area	Height	% Area
1	Peak1 226.0nm	15.365	11381765	586015	49.82
2	Peak2 226.0nm	16.306	11462488	567378	50.18

Chromatogram for racemic **1j** and peak results

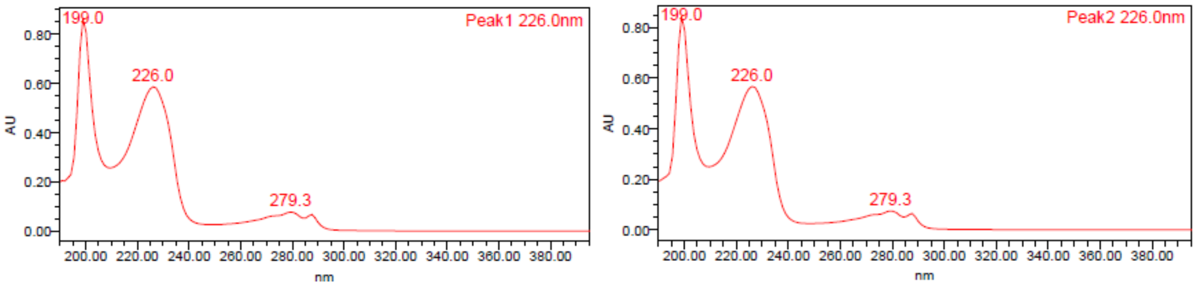

UV spectra at retention times 15,365 and 16,306 respectively

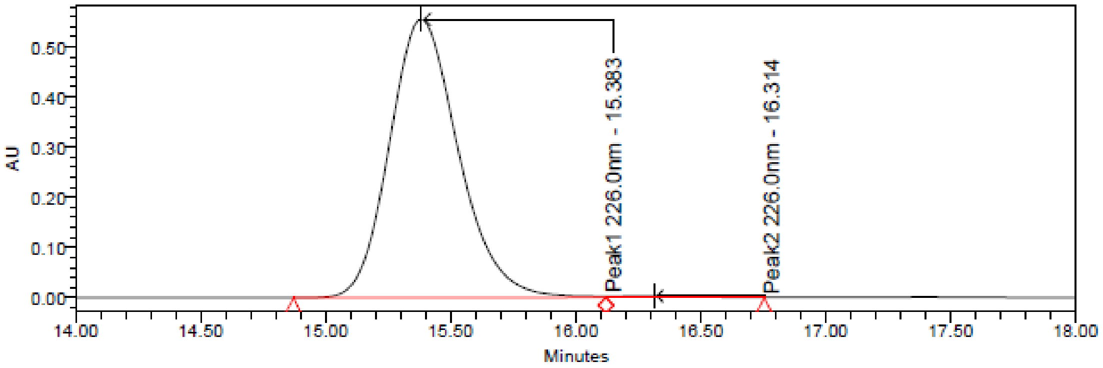

Peak Results

	Name	RT	Area	Height	% Area
1	Peak1 226.0nm	15.383	10666512	555485	99.37
2	Peak2 226.0nm	16.314	67968	3173	0.63

Chromatogram for (*R*)-**1j** and peak results

HPLC Chromatogram for (*R*)-6-chloro-3-iodo-2-methyl-2*H*-chromene (2j)

CHIRALCEL OD-H: *n*-Hexane; 100; Flow 0.6 ml/min; λ = 274.5 nm

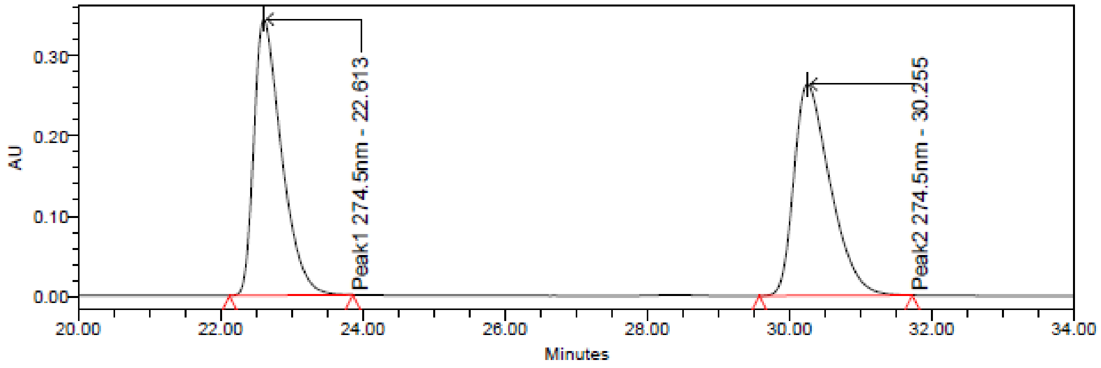

Peak Results

	Name	RT	Area	Height	% Area
1	Peak1 274.5nm	22.613	9288718	343388	49.88
2	Peak2 274.5nm	30.255	9334772	262805	50.12

Chromatogram for racemic **2j** and peak results

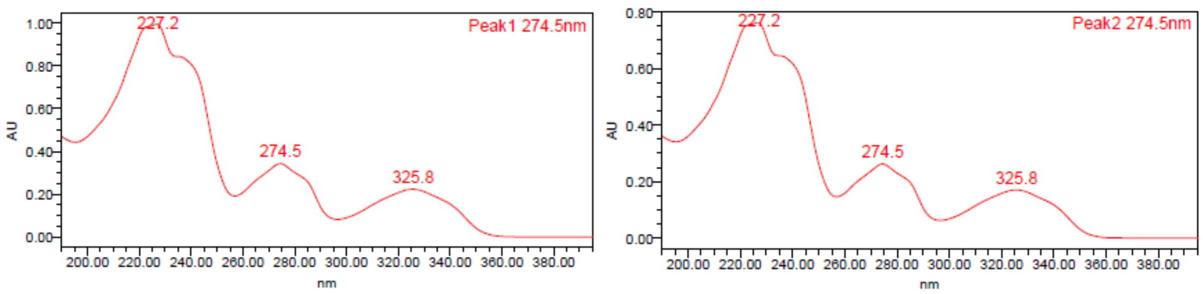

UV spectra at retention times 22,613 and 30,255 respectively

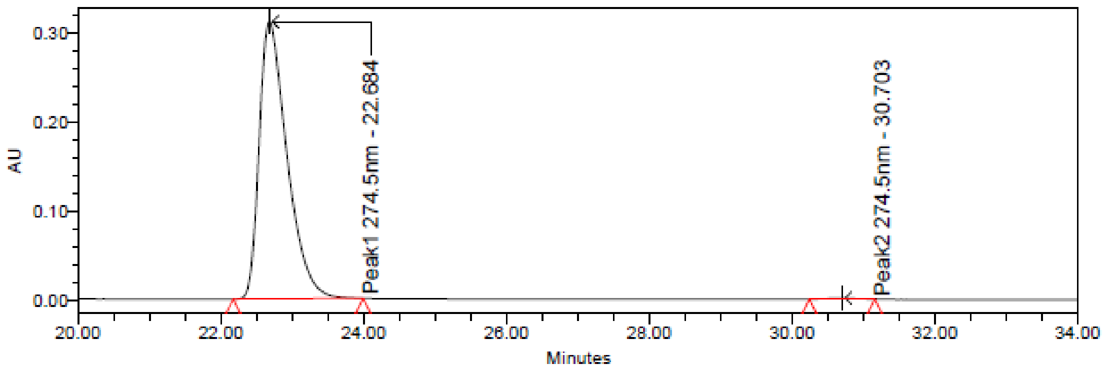

Peak Results

	Name	RT	Area	Height	% Area
1	Peak1 274.5nm	22.684	8421813	311024	99.56
2	Peak2 274.5nm	30.703	37201	1307	0.44

Chromatogram for (*R*)-**2j** and peak results

<u>**5.** Structural assignment for compounds **3**</u>

The products **3** are formed as minor components from the gold-catalyzed cyclization reactions of the starting **1**. Although in general compounds **3** were not isolated from crude reaction mixtures containing compounds **2** as the major regioisomers, some of them were isolated. Thus, below is shown the ^{1}H NMR spectrum for compound **3h**, which was separable from **2h**.

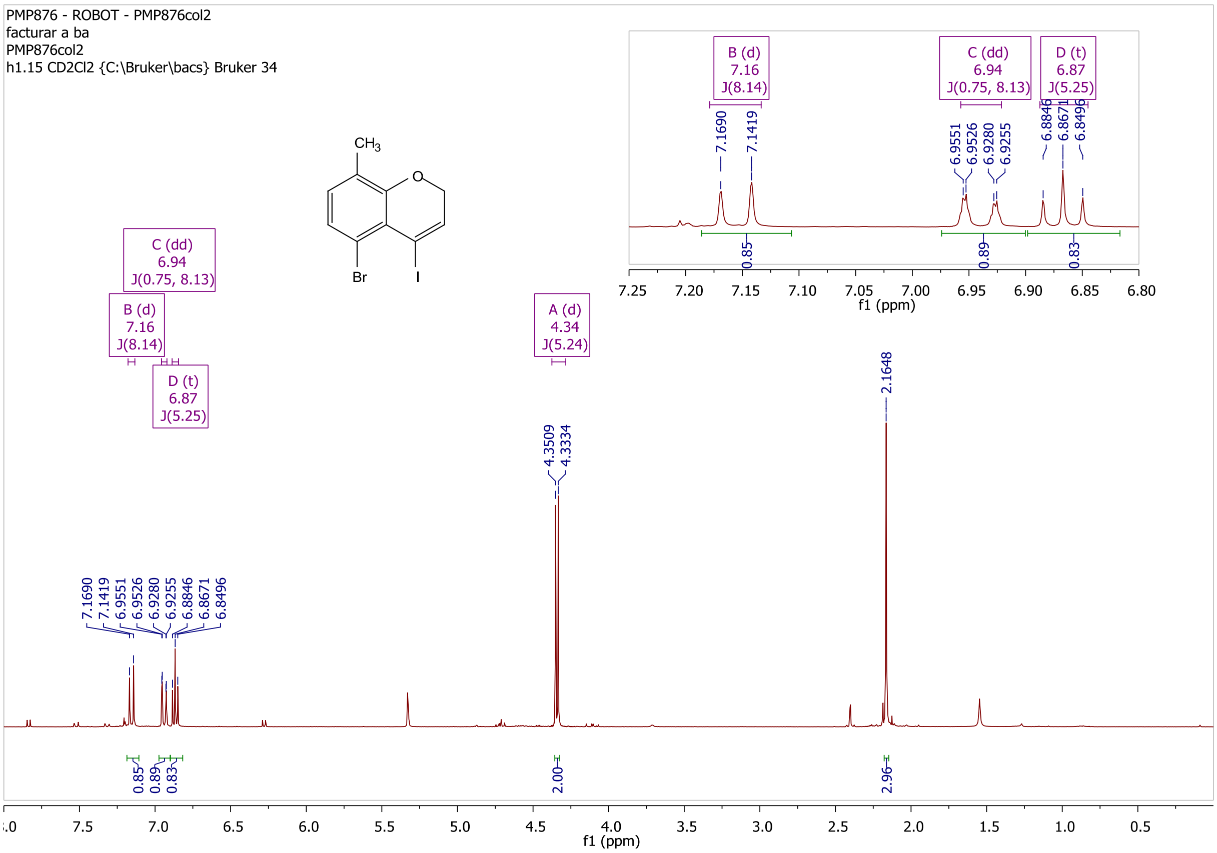

The signals at δ: 4.34 and 6.87 ppm are characteristic for the presence of compounds **3**. They always show coupling constants in the range of 4.5 Hz (higher values than those associated to compounds **2**) and lower chemical shifts than the corresponding signals in compounds **2**. On this basis, regioisomeric ratios were determined from crude reaction mixtures upon inspection by ^{1}H NMR. A representative case is discussed in the next paragraph.

The given ^{1}H NMR corresponds to the reaction depicted in Table 1 for entry 9. It is possible to clearly distinguish and assign the signals for the allylic hydrogens of **2a** (δ = 4.88 ppm; *J* = 1.67 Hz) and **3a** (δ = 4.75 ppm; *J* = 3.98 Hz); and also for the vinylic hydrogen of **2a** (δ = 6.98 ppm; broad singlet) and **3a** (δ = 6.56 ppm; *J* = 3.97 Hz).

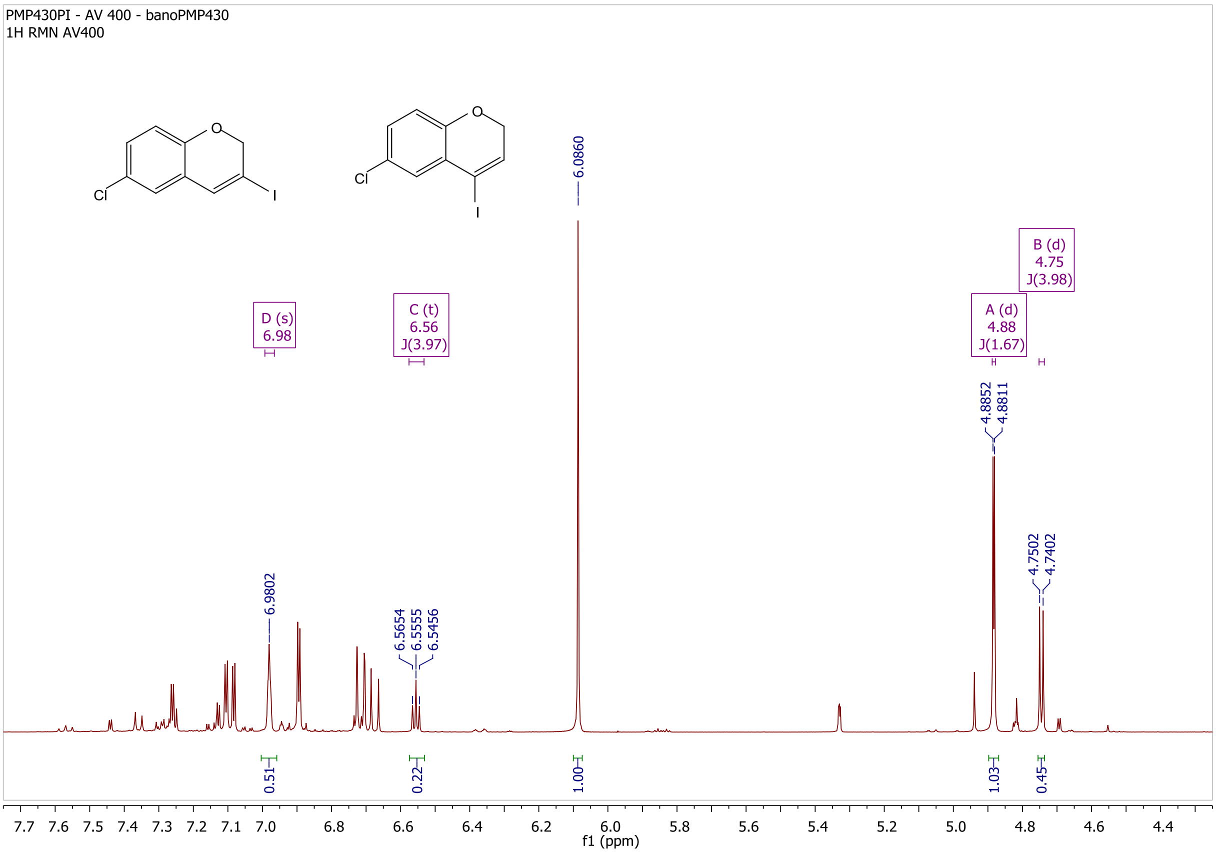
PMP430PI - AV 400 - banoPMP430
1H RMN AV400
Cl
O
I
D (s)
6.98
C (t)
6.56
J(3.97)
A (d)
4.88
J(1.67)
B (d)
4.75
J(3.98)
6.0860
6.9802
6.5654
6.5555
6.5456
4.8852
4.8811
4.7502
4.7402
0.51
0.22
1.00
1.03
0.45
7.7 7.6 7.5 7.4 7.3 7.2 7.1 7.0 6.8 6.6 6.4 6.2 6.0 5.8 5.6 5.4 5.2 5.0 4.8 4.6 4.4
f1 (ppm)
